# Genomic determinants implicated in the glucocorticoid-mediated induction of KLF9 in pulmonary epithelial cells

**DOI:** 10.1074/jbc.RA120.015755

**Published:** 2020-11-23

**Authors:** Mahmoud M. Mostafa, Akanksha Bansal, Aubrey N. Michi, Sarah K. Sasse, David Proud, Anthony N. Gerber, Robert Newton

**Affiliations:** 1 Department of Physiology & Pharmacology and Snyder Institute for Chronic Diseases, Cumming School of Medicine, University of Calgary, Calgary, Canada; 2 Department of Medicine, National Jewish Health, Denver, Colorado, USA; 3 Department of Medicine, University of Colorado, Aurora, Colorado, USA

**Keywords:** airway epithelial cells, air–liquid interface (ALI), gene regulation, chromatin immunoprecipitation (ChIP), enhancer RNA (eRNA), Krüppel-like factor 9 (KLF9), glucocorticoid receptor (GR;NR3C1), CREB binding protein (CBP;CREBBP), E1A binding protein P300 (EP300), ALI, air–liquid interface, CBP, CREB binding protein, ChIP, chromatin immunoprecipitation, CREB, cAMP response element binding protein, C_T_, threshold cycle, eRNA, enhancer RNA, GBS, glucocorticoid receptor binding site, GR, glucocorticoid receptor, GRE, glucocorticoid response elements, GRO-seq, global run-on followed by sequencing, HBE, human bronchial epithelial, KLF, Krüppel-like factor, NHR, nuclear hormone receptor, Pol II, RNA polymerase II, PWM, position weight matrix, qPCR, quantitative polymerase chain reaction, TSS, transcription start site, usRNA, unspliced RNA

## Abstract

Ligand-activated glucocorticoid receptor (GR) elicits variable glucocorticoid-modulated transcriptomes in different cell types. However, some genes, including Krüppel-like factor 9 (KLF9), a putative transcriptional repressor, demonstrate conserved responses. We show that glucocorticoids induce KLF9 expression in the human airways *in vivo* and in differentiated human bronchial epithelial (HBE) cells grown at air–liquid interface (ALI). In A549 and BEAS-2B pulmonary epithelial cells, glucocorticoids induce KLF9 expression with similar kinetics to primary HBE cells in submersion culture. A549 and BEAS-2B ChIP-seq data reveal four common glucocorticoid-induced GR binding sites (GBSs). Two GBSs mapped to the 5ʹ-proximal region relative to *KLF9* transcription start site (TSS) and two occurred at distal sites. These were all confirmed in primary HBE cells. Global run-on (GRO) sequencing indicated robust enhancer RNA (eRNA) production from three of these GBSs in BEAS-2B cells. This was confirmed in A549 cells, plus submersion, and ALI culture of HBE cells. Cloning each GBS into luciferase reporters revealed glucocorticoid-induced activity requiring a glucocorticoid response element (GRE) within each distal GBS. While the proximal GBSs drove modest reporter induction by glucocorticoids, this region exhibited basal eRNA production, RNA polymerase II enrichment, and looping to the TSS, plausibly underlying constitutive KLF9 expression. Post glucocorticoid treatment, interactions between distal and proximal GBSs and the TSS correlated with KLF9 induction. CBP/P300 silencing reduced proximal GBS activity, but negligibly affected KLF9 expression. Overall, a model for glucocorticoid-mediated regulation of KLF9 involving multiple GBSs is depicted. This work unequivocally demonstrates that mechanistic insights gained from cell lines can translate to physiologically relevant systems.

Glucocorticoids are adrenal hormones that play crucial roles in metabolic homeostasis, development, responses to stress, and inflammation (1, 2). Clinically, glucocorticoids are extensively utilized for their often profound, anti-inflammatory actions in treating chronic inflammatory disorders, including asthma, rheumatoid arthritis, and various dermatological diseases (3, 4). Glucocorticoids exert their effects *via* activation of glucocorticoid receptor (GR; official gene symbol NR3C1). Upon ligand binding, GR translocates from the cytoplasm to the nucleus, where it is recruited to thousands of genomic loci and regulates the expression of hundreds of genes (5, 6, 7). Several studies show that the glucocorticoid-dependent recruitment of GR to genomic loci and subsequent regulation of gene expression varies between different tissues, cell types, and cell lines, and is therefore highly context-dependent (8, 9, 10, 11, 12, 13). Nevertheless, many glucocorticoid-induced genes respond to glucocorticoids in a similar fashion regardless of the cell line, type, or tissue of origin (8, 10, 14). While presumably such genes possess genomic characteristics, or features, that are conserved, *i.e.*, common, between different cell types and lines to allow “in common” regulation, this is not generally investigated.

Current models for glucocorticoid-mediated activation of gene expression suggest direct binding of GR to glucocorticoid response elements (GRE), palindromic DNA sequences that may be located 5ʹ, 3ʹ or within the body of target genes (5, 15, 16). Once bound to DNA, glucocorticoid-activated GR recruits cofactors that promote activation, or recruitment and activation, of the transcriptional machinery (17, 18). This increases transcriptional activity at GREs, as evidenced by local RNA polymerase II (Pol II) recruitment and the production of bidirectional enhancer RNA (eRNA), plus leads to directional transcriptional activation across the body of target genes (19, 20). Despite the large number of potential GRE sites throughout the genome, ligand-activated GR only binds to a small fraction of these sites in any given cell type or tissue (21). This is due to the preferential binding of GR to DNA regions that possess features of active enhancers. These features include open chromatin, which strongly suggests occupancy by other transcription factors, the presence of certain histone marks (H3K27 acetylation), and binding of histone acetyltransferases, such as P300 (gene symbol: EP300) (22, 23, 24). Moreover, a considerable fraction of GR binding sites (GBSs) appear to lack strong GRE motifs and exhibit no glucocorticoid-mediated enhancer activation (25, 26). Evidence from transcription factor binding and long-range chromatin interaction studies suggests that such sites may not directly bind GR, but are instead occupied by transcription factors, such as JUN or CEBPB, that interact with GR *via* DNA loops to regions with direct GR binding (7, 24, 27). Thus, models for gene induction by glucocorticoids involve ligand-activated GR orchestrating chromatin interactions that bring transcriptionally active enhancers, including those not necessarily activated by glucocorticoids, into proximity of a target gene’s promoter (27, 28). Accordingly, variability in enhancer accessibility, transcription factor binding, and/or chromatin interactions in different tissues or cell types may produce variable, context-specific regulation of gene expression.

Krüppel-like factor 9 (KLF9), along with 16 other genes, was significantly induced (≥2-fold, *p* ≤ 0.05) by glucocorticoids in three different variants of human pulmonary epithelial cells (8). Such “in common” regulation for KLF9 by glucocorticoids has also been reported in other cell types or tissues. These include primary human lung cells and the human airways *in vivo* (14), adipocytes (29), dental follicle cells (30), hepatocytes (31), keratinocytes (32), macrophages (33, 34), and neuronal cells (35, 36). As KLF9 is a putative transcriptional repressor, whose expression negatively correlated with the ability of cells to proliferate and/or migrate (32, 37, 38), this commonality of expression suggests a fundamental role for KLF9 in the response to glucocorticoids. Nevertheless, while the functional impacts of KLF9 induction remain to be clearly identified, these data also raise the possibility that common mechanisms may lead to KLF9 induction. Unraveling these mechanisms is therefore central to understanding the “in common” induction of KLF9 by glucocorticoids. This is explored below in pulmonary epithelial cell lines and primary cells.

Airway epithelial cells are not only critical in the pathogenesis of asthma, but also represent key effector cells in mediating therapeutic responses to glucocorticoids in dampening airway and lung inflammation (39, 40). The current study therefore takes advantage of the established “in common” glucocorticoid regulation of KLF9 in primary human bronchial epithelial (HBE) cells and both alveolar type II (A549) and bronchial (BEAS-2B) epithelial cell lines (8, 14). These cell lines represent well-studied human models, for which high-quality genomic data exists (5, 19, 20, 24, 26, 27), and are here used to explore genomic features that may impact on the GR-mediated expression of KLF9 in therapeutically relevant cells.

## Results

### Regulation of KLF9 in the human pulmonary epithelial cells

**Figure 1:**
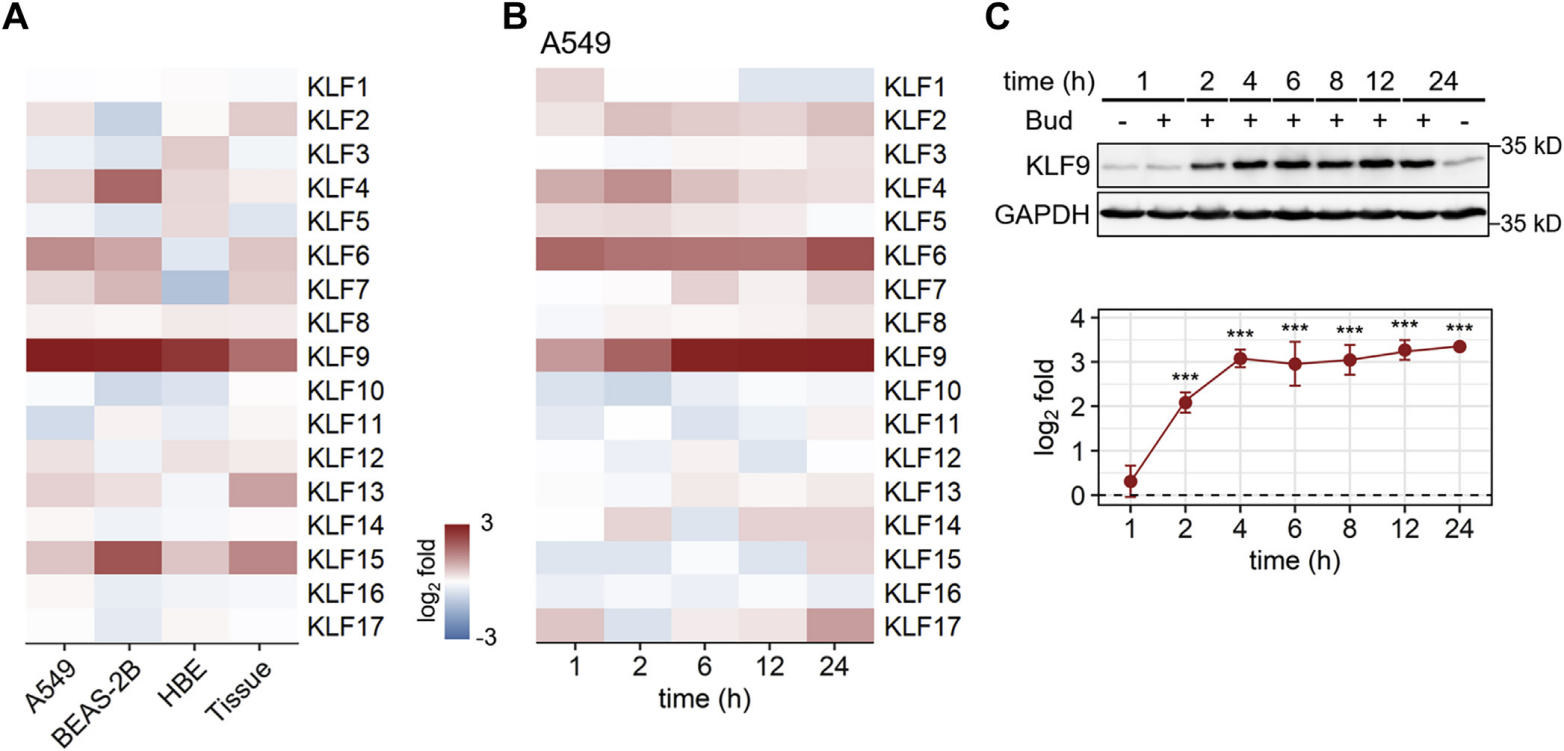
**Effect of budesonide on KLF expression in human epithelial cells and lung tissue.***A*, data were obtained from microarray analyses performed on A549, BEAS-2B, primary human bronchial epithelial (HBE) (8), and human bronchial biopsies (tissue) (14). Cells were exposed to maximally effective concentrations of budesonide (300 nM for A549 and BEAS-2B, or 100 nM for HBE) for 6 h prior to harvest. Bronchial biopsies were collected ∼6 h post high-dose budesonide (1600 μg) inhalation. In each case, the heatmap depicts the effect (log_2_ fold) of budesonide treatment on the expression of the 17 KLFs, as compared with time-matched no treatment control, for cultured cells, or placebo inhalation, for the tissues. *B*, RNA-seq analysis of A549 cells treated with 300 nM budesonide for the indicated time points. The heatmap depicts the effect (log_2_ fold) of budesonide treatment on the expression of the 17 KLFs when compared with no treatment control at each time point according to the same scale as in panel *A*. *C*, A549 cells were either not treated or treated with 300 nM budesonide (Bud) prior to harvesting at the indicated times for western analysis of KLF9 and GAPDH. Representative blots are shown (upper panels). Following densitometric analysis, data (*N* = 4), as KLF9/GAPDH were expressed as log_2_ fold relative to no treatment at 1 h and are plotted as means ± SE (lower panel). Significance, using normalized KLF9/GAPDH values relative to control at 1 h, was tested by ANOVA with Tukey’s post-hoc test. ∗∗∗*p* ≤ 0.001.

Expression data for all 17 KLF genes were extracted from previously published transcriptome analyses of A549, BEAS-2B, and primary HBE cells, each following no stimulation or treatment with maximally effective concentrations of the synthetic glucocorticoid budesonide for 6 h (8). These data were compared with the effect of an inhaled dose (1200 μg) of budesonide in human bronchial biopsies taken 6 h post placebo or budesonide inhalation (14). While various KLFs, including KLF15, revealed modulation by budesonide, only KLF9 showed significant (*p* ≤ 0.05), ≥2-fold, induction by budesonide in all three epithelial cell variants and *in vivo* in airway biopsy tissues (Fig. 1*A*). In addition, comparable increases in KLF9 mRNA expression in response to budesonide were observed in primary human airway smooth muscle (ASM), bronchial fibroblast (HBF), and vascular endothelial (HUVEC) cells (Fig. S1*A*). Thus, not only was the induction of KLF9 mRNA expression by budesonide a conserved response in the airways *in vivo*, as well as in structural cells relevant to the airways, but the effect was recapitulated in the model epithelial cell lines, A549 and BEAS-2B.

In A549 cells treated with budesonide for 1, 2, 6, 12, and 24 h, KLF4 mRNA was modestly (∼2-fold) induced at 1 and 2 h, whereas KLF6 and, to a greater extent, KLF9 were more substantially (≥2-fold) and significantly induced at all times (Fig. 1*B*). Western blotting showed significant increases in KLF9 protein at 2 h and a plateau of peak expression that extended from 4 to 24 h (Fig. 1*C*). Similarly, transcriptomic data from BEAS-2B cells revealed significant (*p* ≤ 0.05) ≥2-fold induction of KLF4, KLF6, and KLF7 mRNAs by budesonide with peak expression occurring at 1 or 2 h (Fig. S1*B*) (8). KLF15 mRNA also showed significant, ≥2-fold budesonide-induced expression that peaked at 6 h. KLF9 mRNA revealed peak expression at 2 h, but was significantly induced, ≥2-fold, at all times (1, 2, 6, and 18 h) analyzed. Western blotting indicated increased KLF9 protein from 1 h post budesonide treatment of BEAS-2B cells that peaked at 6 h and remained highly elevated at 18 h (Fig. S1*C*).

Several studies have reported KLF9 regulation by various nuclear hormone receptor (NHR) agonists, including thyroxine, progesterone, and estrogen (reviewed in (41)). To assess their influence on KLF9 expression, A549 cells were treated with agonists of the glucocorticoid (GR), progesterone (PGR), estrogen (ESR), thyroxine (THRs), vitamin D (VDR), retinoic acid (RARs), retinoid (RXRs), RAR-related (RORs), or Rev-Erb (NR1Ds) receptors (Fig. S1*D*). Budesonide and triiodo-L-thyronine, agonists at GR and THRs, induced KLF9 mRNA expression by 6.9 and 2.8-fold, respectively (both *p* ≤ 0.05). Dihydroxyvitamin D3, a VDR agonist, also induced KLF9 expression by 2.2-fold, but this effect was variable and did not reach significance. Whereas other NHR ligands were largely without effect, the above data indicate glucocorticoids, presumably acting through GR, to be a major regulator of KLF9 expression in the lungs, including the epithelium, as modeled by A549 cells.

**Figure 2:**
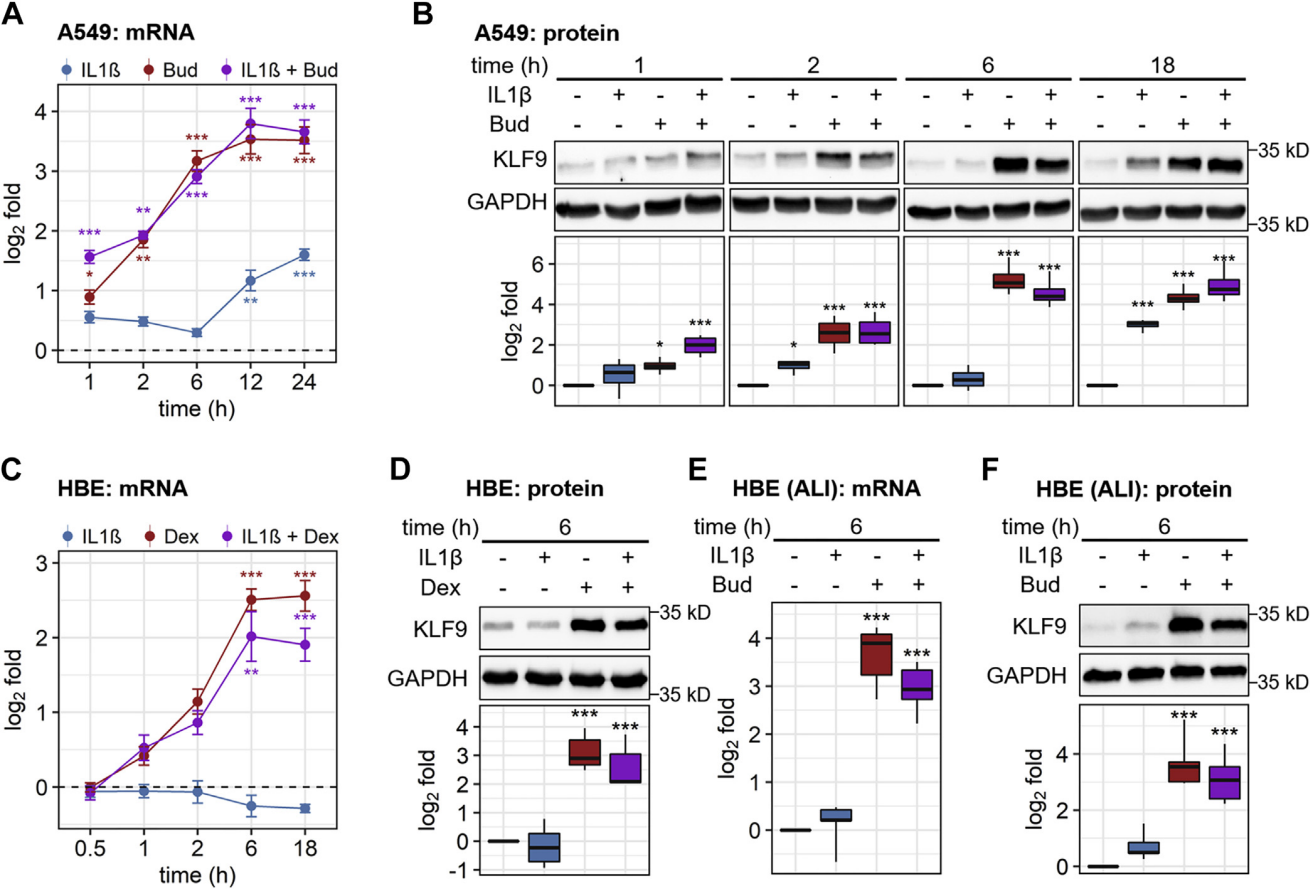
**Regulation of KLF9 expression by glucocorticoids and/or IL1B in human pulmonary epithelial cell models.** A549 cells (*A*–*B*) and primary human bronchial epithelial (HBE) cells grown in submersion culture (*C*–*D*), or air–liquid interface (ALI) culture (*E*–*F*) were either not treated or treated with IL1B (1 ng/ml), glucocorticoid (300 nM budesonide (Bud) or 1 μM dexamethasone (Dex)), or the combination for the indicated times. *A*, *C*, and *E*, cells were harvested for RNA and qPCR was performed for KLF9 and GAPDH. Data (*N* = 4–5), expressed as KLF9/GAPDH, were plotted as log_2_ fold relative to no treatment control at each time point. *B*, *D*, and *F*, cells were harvested for western blot analysis of KLF9 and GAPDH. Representative blots are shown (upper panels). Following densitometric analysis, data (*N* = 4–5), as KLF9/GAPDH were expressed as log_2_ fold relative to no treatment and are plotted as means ± SE (*A* and *C*), or box-and-whiskers plots (*B*, *D*, *E*, and *F*). Significance, using normalized KLF9/GAPDH values relative to no treatment control at each time, was tested by ANOVA with Tukey’s post-hoc test. ∗*p* ≤ 0.05, ∗∗*p* ≤ 0.01, ∗∗∗*p* ≤ 0.001.

While glucocorticoids are used for their anti-inflammatory effects, a significant fraction of the glucocorticoid-regulated transcriptome is modulated by inflammatory stimuli, including *lipopolysaccharide* (LPS) and TNF (20, 33, 42, 43). A549 cells were therefore treated with budesonide, interleukin-1β (IL1B), or their combination for 1, 2, 6, 12, and 24 h (Fig. 2*A*). KLF9 mRNA was significantly induced by budesonide at 1 h and showed a plateau of peak expression that extended from 6 to 24 h. Alone, IL1B significantly induced KLF9 mRNA expression at 12 and 24 h (Fig. 2*A*). In combination with budesonide, IL1B enhanced the effect of budesonide at 1 h, but did not modify inducibility by budesonide at other times. Western blotting of A549 cell lysates showed significant induction of KLF9 protein by IL1B at 18 h, as well as confirmed the slightly enhanced inducibility by budesonide in the presence of IL1B at 1 h (Fig. 2*B*). In primary HBE cells, a maximally effective concentration of dexamethasone, which was previously found to be synonymous with that for budesonide in A549 cells (8), also resulted in similar responses. Thus, dexamethasone modestly enhanced KLF9 mRNA at 1 and 2 h (Fig. 2*C*). This reached a peak at 6 h and remained significantly elevated at 18 h posttreatment (Fig. 2*C*). Alone, IL1B modestly, but nonsignificantly, decreased KLF9 expression at 6 and 18 h in the HBE cells. While IL1B exerted little effect on the initial induction of KLF9 mRNA by dexamethasone, by 18 h a modest, but nonsignificant, repressive effect was apparent (Fig. 2*C*). Western blotting confirmed induction of KLF9 protein in HBE cells at 6 h by dexamethasone with no clear effect of IL1B (Fig. 2*D*). Similarly, in highly differentiated primary HBE cells grown at air–liquid interface (ALI), KLF9 mRNA and protein were induced by budesonide at 6 h, and there was no significant impact of IL1B (Fig. 2, *E*–*F*). Thus, KLF9 mRNA and protein were robustly induced by glucocorticoids in A549 and primary human HBE cells grown in submersion and ALI culture, and there was little effect of IL1B on induction of KLF9 by glucocorticoids.

### GR binds to and activates transcription from multiple enhancer elements upstream of KLF9

**Figure 3:**
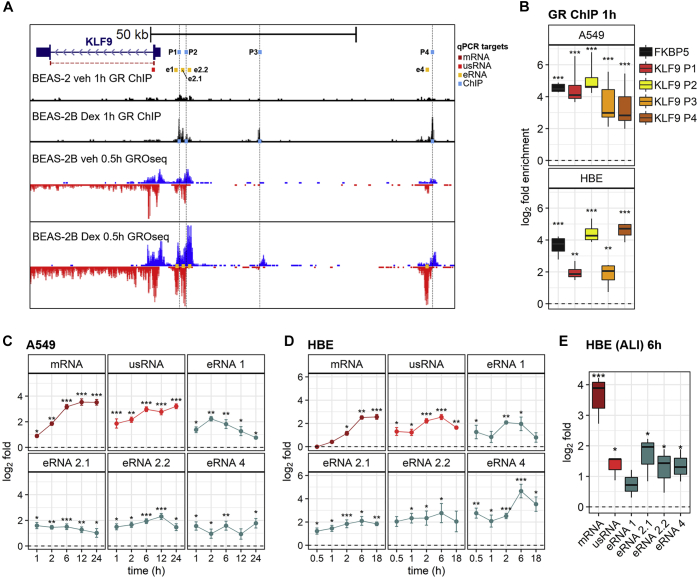
**GR binding and transcriptional activity at the *KLF9* locus following glucocorticoid treatment.***A*, genome browser snapshot of the *KLF9* gene along with ∼70 kb upstream of *KLF9*. Arrow heads within the single intron indicate direction of transcription. GR ChIP-seq traces following a 1 h treatment of BEAS-2B cells with vehicle control (veh) or 100 nM dexamethasone (Dex) are shown (data from Kadiyala *et al.*, 2016 (19); upper two tracks). GRO-seq data showing nascent RNA mapped to the same region upon vehicle or Dex treatment, as above, for 30 min in BEAS-2B cells are shown (data from Sasse *et al.*, 2019 (20); lower two tracks). Nascent transcripts mapped to the (+) or (−) strands are shown in *blue* or *red*, respectively. Approximate positions of qPCR amplicons for the detection of GR ChIP peaks (*light blue*), mRNA (*dark red*), unspliced RNA (usRNA) (*bright red*), and enhancer RNA (eRNA) (*orange*) are highlighted. *B*, A549 or primary HBE cells were either not stimulated or treated with 300 nM budesonide for 1 h prior to ChIP-PCR for GR. PCR primers were designed to span an intronic GR binding site (GBS) in the *FKBP5* gene (positive control), as well as the four GBSs upstream of *KLF9*; P1, P2, P3, and P4 (*light blue boxes* in *A*). PCR data were normalized to the geometric mean of three control regions that are not occupied by GR. Normalized data, *N* = 3 to 4 (performed with two technical replicates), were plotted as log_2_ fold enrichment relative to no treatment control. A549 cells (*C*), primary HBE cells in submersion culture (*D*), or primary HBE cells in ALI culture (*E*) were either not stimulated or treated with glucocorticoid (300 nM budesonide (*C* and *E*) or 1 μM dexamethasone (*D*)) for the indicated times prior to qPCR analysis of: mature KLF9 mRNA, KLF9 usRNA, and the four eRNAs, e1, e2.1, e2.2, and e4. Primers are as indicated in panel *A*. For normalization, GAPDH (for mRNA) and U6 (for usRNA and eRNAs) were also assayed. Normalized data (*N* = 3–4) were plotted as log_2_ fold relative to not stimulated control at each time point. Data are shown as box-and-whiskers plots (*B* and *E*) or as means ± SE (*C*–*D*). Significance, using normalized data relative to no treatment control at each time, was tested by paired *t* test. ∗*p* ≤ 0.05, ∗∗*p* ≤ 0.01, ∗∗∗*p* ≤ 0.001.

To investigate mechanisms by which glucocorticoids regulate KLF9 expression, GR binding around the *KLF9* locus was examined using previously published ChIP-seq data from BEAS-2B cells following dexamethasone treatment (19). After 1 h of dexamethasone treatment, multiple GBSs were clearly apparent that ranged from <10 kb upstream of the transcription start site (TSS) to greater than 50 kb from the TSS (Fig. 3*A*, Fig. S2*A*). A very similar pattern of GBS localization was also observed in dexamethasone-treated A549 cells (Fig. S2*A*) (24). Since no other glucocorticoid-induced genes were in the immediate vicinity, roles for these GBSs in the regulation of KLF9 expression were considered plausible (Fig. S2*B*). ChIP-PCR primers were therefore designed for two proximal and two distal KLF9 GBSs that were common to both A549 and BEAS-2B cells (Fig. S2*A*). These sites were located 5.9 (P1), 6.7 (P2), 25 (P3), and 65 (P4) kb upstream of the *KLF9* gene (Fig. 3*A*, upper tracks). In A549 cells, ChIP-PCR showed that 1 h treatment with budesonide produced robust and significantly increased GR enrichments at each of these four regions (Fig. 3*B*, upper panel). For regions P1 and P2, GR enrichment was to similar level as that found for a previously described intronic GBS located in the *FKBP5* gene and which served as a strong positive control (8, 19, 44, 45). ChIP-PCR in primary HBE cells similarly revealed significant budesonide-induced GR enrichment (or occupancy) at all four regions (Fig. 3*B*, lower panel).

To visualize transcriptional activity arising from the *KLF9* locus, data from a previously published global run-on (GRO)-seq experiment in BEAS-2B cells treated with vehicle or dexamethasone for 0.5 h are displayed (Fig. 3*A*) (20). Compared with vehicle, dexamethasone enhanced nascent transcript production throughout the body of the *KLF9* gene. Note that the *KLF9* gene is orientated in a negative sense relative to the reference human genome (GRCh38), and this nomenclature is maintained throughout the current analysis. Dexamethasone treatment also increased bidirectional production of nascent RNAs from the *KLF9* TSS as well as from the four (P1-P4) upstream GBSs (Fig. 3*A*, lower tracks). These latter transcripts, which are consistent with a formal definition of enhancer RNAs (eRNAs) (20, 46), are hereafter referred to as eRNA 1 to 4, depending on the associated GBS. In each case, qPCR primers were designed to amplify eRNAs in the P1/P2, P3, and P4 regions (orange boxes in Fig. 3*A*). While amplicons for eRNAs 1, 2.1, 2.2, and 4 were readily detected following glucocorticoid treatment, detection of the P3 eRNA was not possible. Expression of eRNAs 1, 2.1, 2.2, and 4, as well as unspliced/unprocessed KLF9 transcript (usRNA), as a surrogate for transcription rate, was analyzed in A549 and primary HBE cells following either budesonide or dexamethasone treatment, respectively (Fig. 3, *C*–*D*). As shown above, induction of KLF9 mRNA expression by glucocorticoid in A549 cells was initially low at 1 h, before robustly increasing at 2 h and reaching a peak from 6 h onward (Fig. 3*C*). A similar effect was also apparent in HBE cells, except that mRNA inducibility was undetectable at 30 min and less than 1.5-fold at 1 h, prior to increasing with near maximal expression observed from 6 h onward (Fig. 3*D*). These data contrast with KLF9 transcription rate as assessed by the accumulation of KLF9 usRNA. At 1 h in A549 cells, or 30 min in HBE cells, the fold induction for KLF9 usRNA was 4 and 2.7-fold, respectively. In A549 cells, this further increased to a plateau of 8- to 9.5-fold that spanned from 6 to 24 h. A similar effect was found in HBE cells. Thus, KLF9 transcription was rapidly (within 1 h) increased by glucocorticoid and this preceded mature mRNA formation. After this initial rapid rise in KLF9 transcription, a secondary increase led to near peak, or peak, transcription occurring at 6 h in both systems.

Similar to KLF9 usRNA, eRNAs from the four regions tested were significantly induced by glucocorticoid at the earliest time points in both A549 and HBE cells (Fig. 3, *C*–*D*). Furthermore, while some variations in expression pattern were observed for the different eRNAs, their induction was largely maintained at similarly elevated levels for the duration of the experiment (Fig. 3, *C*–*D*, Fig. S3, *A*–*B*). Notably, baseline expression of eRNAs 1, 2.1, and 2.2 was higher than for eRNA 4 (Fig. S3, *A*–*B*). This was observed in both A549 and primary HBE cells and is consistent with both the relatively strong GRO-seq signal and the presence of Pol II at the P1/P2 region compared with P3 and P4 regions in vehicle-treated BEAS-2B cells (Fig. 3*A*, Fig. S3*C*). In primary HBE cells grown in ALI culture, KLF9 usRNA and eRNAs 2.1, 2.2, and 4 were all significantly induced by budesonide at 6 h (Fig. 3*E*). Likewise, eRNA 1 was also induced, but this failed to reach significance. Nevertheless, these data collectively indicate that prolonged induction of KLF9 by glucocorticoids is associated with sustained transcriptional activation involving multiple GBSs at the *KLF9* locus. The findings also suggest that baseline expression of KLF9 is associated with ongoing transcriptional activity involving the P1/P2 region, even in the absence of glucocorticoid.

### KLF9 induction by glucocorticoids is GR dependent

**Figure 4:**
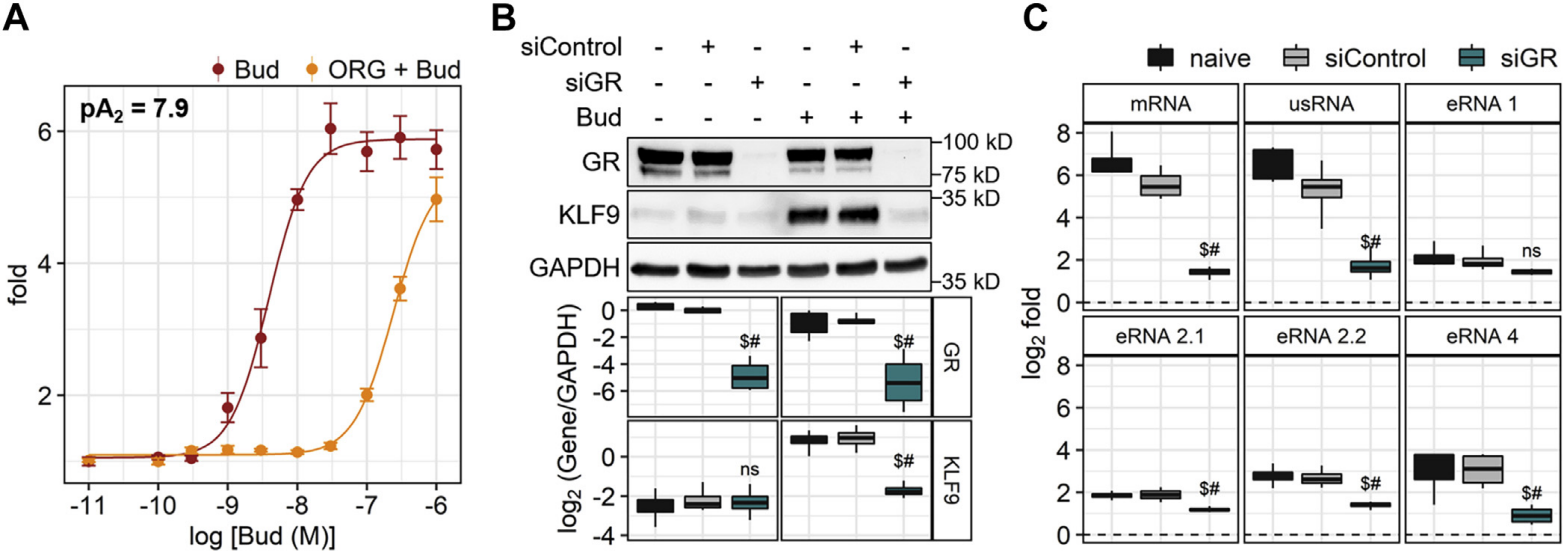
**Glucocorticoid-mediated induction of KLF9 is GR-dependent.***A*, A549 cells were either not stimulated or treated with 1 μM of GR antagonist, Org34517 (ORG), for 30 min prior to stimulation with increasing concentrations of budesonide (Bud), as indicated. After 6 h, cells were harvested for RNA and qPCR was performed for KLF9 and GAPDH. Data (*N* = 4), expressed as KLF9/GAPDH, were plotted as fold change relative to no treatment control and concentration-response curves were constructed. *B*–*C*, A549 cells were incubated with transfection lipid alone (naïve; *black*), or lipid plus 1 nM of nontargeting siRNA (siControl; *gray*) or 1 nM of a pool of four siRNAs against GR (siGR; *green*) for 36 h. Cells were then either not stimulated or treated by 300 nM budesonide (Bud) for 6 h. *B*, cells were harvested for western blot analysis of GR, KLF9, and GAPDH. Representative blots are shown (*upper panel*). Following densitometric analysis, data (*N* = 4), were expressed as log_2_ of the normalization product (gene/GAPDH). *C*, cells were harvested for RNA and qPCR was performed for KLF9 mRNA, KLF9 usRNA, and the four eRNAs, 1, 2.1, 2.2, and 4. For normalization, GAPDH and U6 were also assayed. Normalized data (*N* = 4), as mRNA/GAPDH, usRNA/U6, or eRNA/U6, were plotted as log_2_ fold relative to no treatment of each condition (naïve, siControl, or siGR). *B*–*C*, significance (*p* ≤ 0.05), using normalized data relative to naïve ($) or siControl (#) groups, was tested by ANOVA with Tukey’s post hoc test. Data are shown as mean ± SE (*A*) or as box-and-whiskers plots (*B*–*C*). “ns” = not significant.

In A549 cells, budesonide increased the expression of KLF9 in a concentration-dependent manner that was competitively inhibited by the GR antagonist, Org34517 (Fig. 4*A*). Schild analysis produced a p*A*_2_ of 7.9, which is consistent with antagonism of GR-mediated responses (45, 47, 48). In addition, siRNA-mediated knockdown of GR produced a ∼95% loss of budesonide-induced GR protein (Fig. 4*B*). At the RNA level, GR knockdown significantly reduced induction of KLF9 mRNA, usRNA, and three of the four eRNAs by budesonide (Fig. 4*C*). Inducibility of eRNA 1 by budesonide was also reduced, but this did not reach significance. These data confirm a role for GR in the glucocorticoid-mediated induction of KLF9 mRNA, KLF9 usRNA, and eRNAs derived from the *KLF9* locus.

### Glucocorticoids enhance reporter activity of isolated GBSs

**Figure 5:**
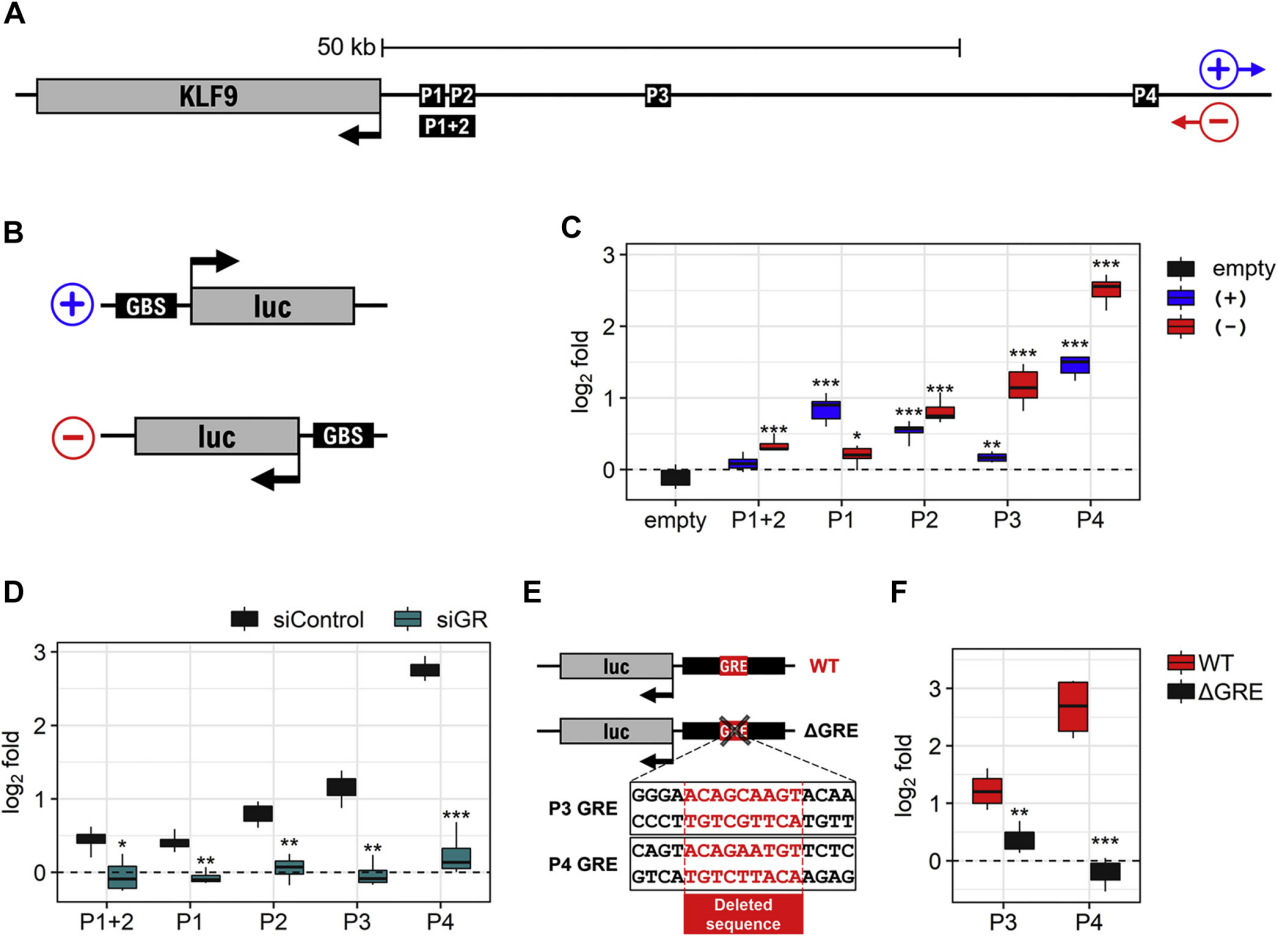
**Reporter activity of isolated KLF9 GBSs.***A*, schematic of the *KLF9* gene along with the ∼70 kb upstream region harboring the four GBSs described in Figure 3. For consistency, the default GRCh38 convention for positive (+) and negative (‒) strand orientation was adopted, where the *KLF9* gene is shown as a (‒) strand gene. *Black boxes* indicate approximate positions of KLF9 GBSs that were PCR-amplified prior to cloning upstream of a luciferase reporter. *B*, schematic illustrating the orientation of the cloned GBSs upstream of a luciferase reporter, where (+) resembles enhancer activity away from the *KLF9* locus and (‒) resembles enhancer activity toward the *KLF9* locus. *C*, A549 cells stably transfected with empty vector (*black*) or reporter constructs for the (+) (*blue*) or (‒) (*red*) orientations of the P1+2, P1, P2, P3, and P4 GBS regions (as described in *A*–*B*) were either not treated or treated with 300 nM of budesonide for 6 h prior to luciferase assay. Data (*N* = 4), expressed as log_2_ fold relative to no treatment control, are plotted as box-and-whiskers. Significance, using RLU values relative to no treatment control in each reporter cells, was tested by paired *t* test. *D*, A549 cells stably transfected with empty vector or reporter constructs for the (‒) orientation of the P1+2, P1, P2, P3, and P4 GBSs were incubated with either 1 nM of a pool of 4 nontargeting siRNAs (siControl; *black*) or 1 nM of a pool of 4 siRNAs targeting GR (siGR; *green*) for 48 h. Cells were then either not treated or treated with 300 nM budesonide for 6 h prior to luciferase assay. Data (*N* = 4), normalized as RLU/RLU_empty_ for each condition (siControl or siGR), were expressed as log_2_ fold relative to no treatment control and are plotted as box-and-whiskers. Significance, using fold change values relative to that of siControl group, was tested by unpaired *t*-test. *E*, schematic showing deletions generated in the P3 and P4 glucocorticoid response elements (GRE). *F*, A549 cells stably transfected with reporter constructs for P3(‒) or P4(‒) GBSs harboring wild-type GRE (WT; *red*) or with deleted GRE (ΔGRE; *black*) were either not stimulated or treated with 300 nM budesonide for 6 h prior to luciferase assay. Data (*N* = 4) expressed as log_2_ fold relative to no treatment control are plotted as box-and-whiskers. Significance, using fold change relative to that of WT constructs, was tested by unpaired *t* test. ∗*p* ≤ 0.05, ∗∗*p* ≤ 0.01, ∗∗∗*p* ≤ 0.001.

Transcriptional activity from each of the four KLF9 GBSs was assessed in isolation of its genomic context. Each GBS, with its flanking DNA (300 to 700 bp), as well as the larger region harboring the P1 and P2 sites (P1+2; ∼1300 bp), was cloned upstream of a luciferase reporter in an orientation that either matched the native orientation of the *KLF9* gene (−strand) or was reversed (+strand) (Fig. 5, *A*–*B*). Stably transfected A549 cells were established for each reporter plasmid as well as the empty vector. In the absence of budesonide, both orientations of the P1+2, P1, and P2 reporters showed 10- to 200-fold higher baseline reporter activity compared with cells stably transfected with the empty vector (Fig. S3*D*). This effect was also evident with the P3(+), but not P3(−), reporter (Fig. S3*D*). With the exception of P1+2(+) and the empty vector, these reporters showed significant induction of luciferase activity by budesonide (Fig. 5*C*). However, while the P4(−) reporter produced approximately 6-fold inducibility by budesonide, ≥2-fold inducibility was achieved by the P3(−) and P4(+) reporters.

Using the P1+P2(−) and P1−P4(−) reporters, siRNA targeted to GR, but not control siRNA, abolished the glucocorticoid-mediated induction of each reporter without affecting basal reporter activity (Fig. 5*D*, Fig. S3*E*). Thus, GR is necessary for glucocorticoid-mediated activation of these reporters, but plays no role in basal activity.

Using the JASPAR CORE database (49), transcription factor binding motif analysis on each of the KLF9 GBSs identified a single strong GRE at the center of each GBS (Fig. S4). In each case, the similarity score compared with the GRE position weight matrix (PWM) was >400, with the GRE at the center of the P4 GBS having the highest score at 609 (Fig. S4, *B*–*C*). Such scores are calculated based on the probability that the candidate sequence matches the GRE PWM, where 0 corresponds to a *p* value of 1 and 1000 to a *p* value ≤10^−10^ (49, 50). A score of 400 (*i.e.*, *p* value of 10^−4^) represents the default cutoff suggested by JASPAR for reliable prediction of transcription factor binding sites. Scores obtained for the GRE motifs within each of the *KLF9* GBSs were therefore consistent with GR directly binding to these sites. To explore the role of these sites in driving glucocorticoid-mediated transcription, the putative GRE sequences at the center of the two most inducible constructs, P3(−) and P4(−), were deleted prior to stable transfection into A549 cells (Fig. 5*E*). In each case, induction of reporter activity by budesonide was profoundly inhibited when compared with the wild-type (WT) constructs (Fig. 5*F*). Thus, each GRE sequence was essential for glucocorticoid-mediated induction of reporter activity. As silencing of GR led to similar outcomes, these data support the conclusion that ligand-activated GR interacts directly with these GREs to promote enhancer activity.

### Long-range chromatin interactions occur between KLF9 GBSs

**Figure 6:**
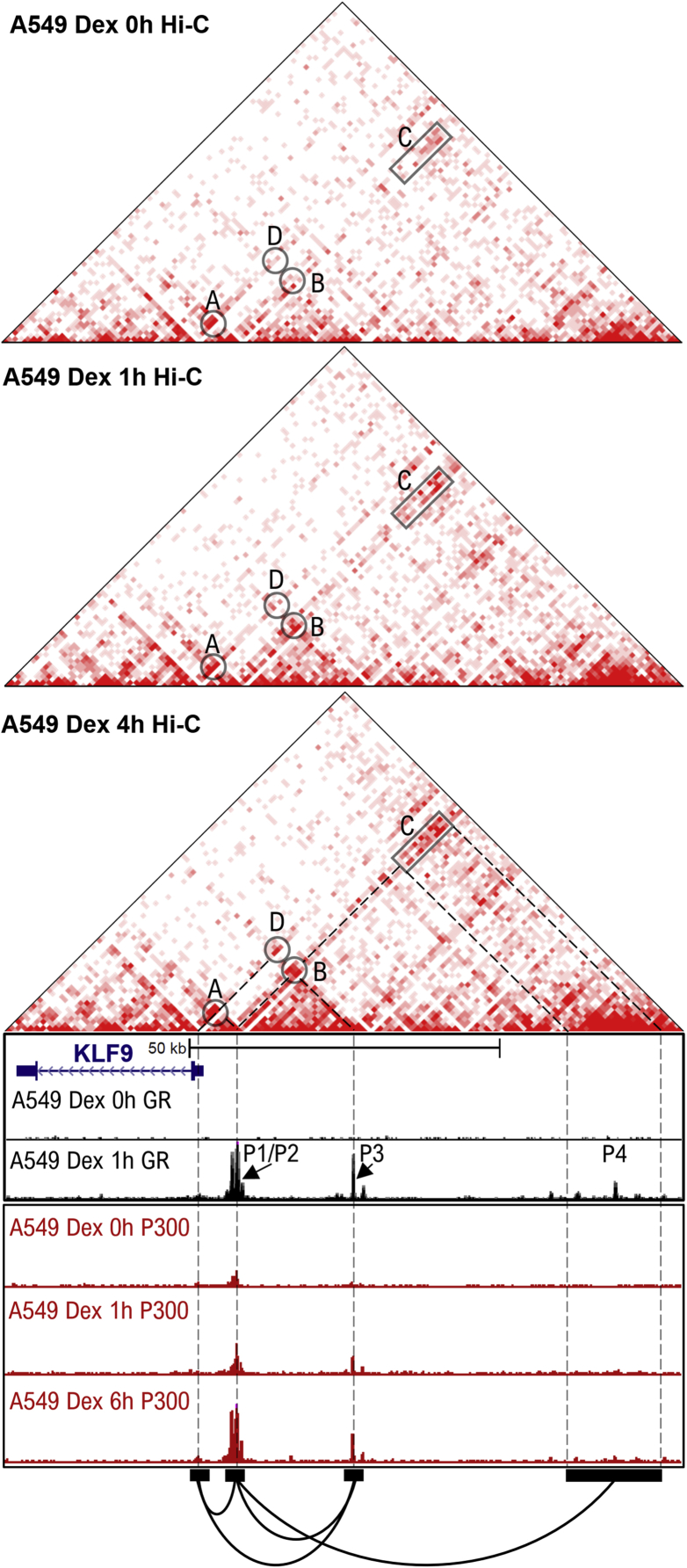
**Interaction between upstream GBSs and the *KLF9* TSS in the absence and presence of glucocorticoid.** Hi-C analysis (unbiased, genome-wide chromosome conformation capture) of untreated A549 cells or following 100 nM Dex treatment for 1 or 4 h is displayed as interaction matrices (*upper three triangular panels*). These correspond to the *KLF9* locus and upstream regions containing GBS P1-4. Images were generated from publicly accessible data from Genomics of Gene Regulation (GGR) project (27). The intensity of the red color reflects the number of ligation events and is indicative of the interaction frequency between the two respective genomic loci joined by downward 45° sloping lines. Interaction hotspots (*A*–*D*) involving the four KLF9 GBSs (P1-4) and TSS are annotated and are linked with dashed lines (*lower panels*). Genome browser snapshot of the *KLF9* gene along with ∼70 kb of the 5′ upstream region showing ChIP-seq traces for GR (*black*) and P300 (*red*) in A549 cells following treatment with 100 nM dexamethasone (Dex) for indicated times. Positions of KLF9 GBSs P1-4 are indicated. ChIP-seq traces are publicly accessible data from GGR project (24, 27).

Recently published work from the Genomics of Gene Regulation (GGR) project described genome-wide chromatin interactions (using *in situ* Hi-C) and transcription factor binding (using ChIP-seq) following dexamethasone treatment of A549 cells (27). Using these data, chromatin interaction matrices corresponding to the region around the *KLF9* gene were visualized using the “Juicebox” webtool (51) (Fig. 6). The color intensity at any point in the matrix reflects the number of ligation events and is indicative of the interaction frequency between the two respective genomic loci linked by downward 45° sloping lines from any point. Thus, interaction hotspots involving the four *KLF9* GBSs (P1-4) and TSS were annotated A to D (Fig. 6). In the absence of dexamethasone, there was a relatively high frequency of interaction between the *KLF9* TSS region and the P1/P2 region (hotspot A), and this was not overly increased following dexamethasone treatment. However, dexamethasone time-dependently increased interactions involving the P1/P2 region with the P3 and P4 regions (Fig. 6; hotspots B and C). In addition, dexamethasone promoted interaction between the P3 region and the *KLF9* TSS region (hotspot D). Collectively, these data indicate baseline interactions between the P1/P2 and *KLF9* TSS regions (hotspot A), which supports a role for the P1/P2 region in basal KLF9 expression in the absence of glucocorticoid. As the P1/P2 region also anchors the dexamethasone-enhanced interactions with the P3 and P4 GBSs, this region may be fundamental for integrating glucocorticoid-mediated transcriptional activation originating from the more distant GBSs, P3 and P4.

D’Ippolito and coworkers also assessed the impact of glucocorticoid on the genomic binding of P300, a transcriptional cofactor with histone acetyltransferase activity that, along with its closely related paralog, CREB-binding protein (CBP) (gene symbol CREBBP) is believed to play a role in glucocorticoid-mediated transcription (28, 52, 53, 54, 55). In the absence of dexamethasone, P300 was enriched at the P1/P2 region (Fig. 6, lower tracks). This was time-dependently increased following dexamethasone treatment and reached a peak 6 h posttreatment. Similarly, dexamethasone time-dependently recruited P300 to the P3 region. These data suggest a possible role for P300 both in the maintenance of basal KLF9 expression through constitutive effects at P1/P2 and in glucocorticoid-mediated induction of KLF9 transcription *via* enhanced recruitment to P1/P2 and P3. Notably, H3K27Ac ChIP-seq from the same study showed enrichment of H3K27Ac marks at *KLF9* promotor and around P1/P2 region in the absence of dexamethasone (Fig. S5) (24). This is consistent with the P1/P2 region being transcriptionally active at baseline, as noted above. Following dexamethasone treatment, the increased enrichment of P300 at P3 region was associated with a gain of the H3K27Ac mark. Nevertheless, this was not observed at the P1/P2 region, which witnessed a slight decrease in H3K27Ac enrichment despite the increase in P300 recruitment (Fig. 6, Fig. S5). The P4 region showed a modest increase in H3K27Ac enrichment following dexamethasone treatment. Collectively, these data indicate that P1/P2 region possesses features of active enhancers at baseline, while P3 and P4 regions gain P300 recruitment and/or H3K27Ac marks following glucocorticoid treatment.

### Transcriptional cofactors CBP and P300 are not required for glucocorticoid-mediated induction of KLF9

**Figure 7:**
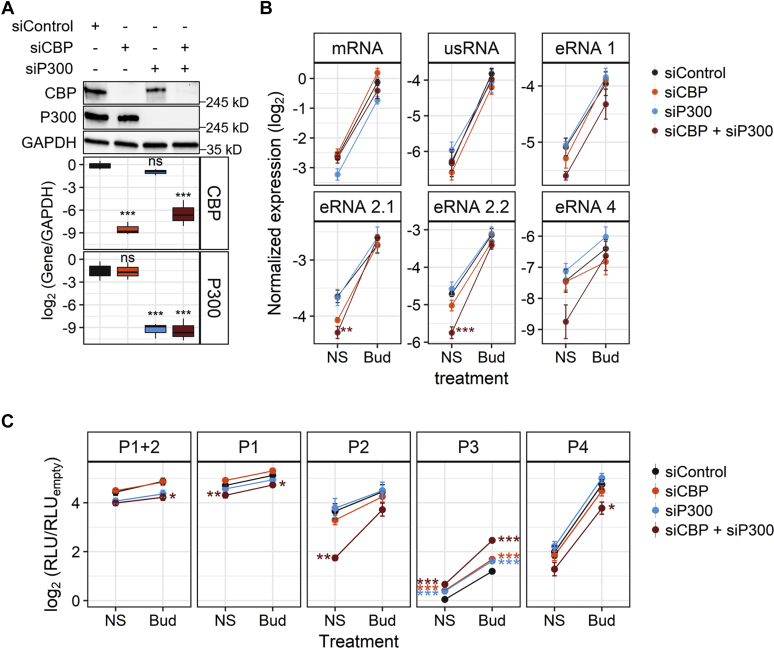
**Effect of CBP and P300 knockdown on KLF9 expression and enhancer activity from KLF9 GBSs.***A*–*B*, A549 cells were incubated with pools of four siRNAs for 48 h. The pools were either nontargeting siRNA (siControl; *black*), or siRNAs targeted to CBP (siCBP; *orange*), P300 (siP300; *light blue*), or combined CBP/P300 siRNAs (*dark red*). *A*, cells were harvested for western blot analysis of CBP, P300, and GAPDH. Representative blots are shown (*upper panel*). Following densitometric analysis, normalized data (*N* = 4), expressed as log_2_ (gene/GAPDH), are plotted as box-and-whiskers plots. *B*, cells were either not stimulated (NS) or treated with 300 nM budesonide (Bud) for 6 h prior to qPCR analysis of: mature KLF9 mRNA, KLF9 usRNA, and the four eRNAs, 1, 2.1, 2.2, and 4. For normalization, GAPDH and U6 were also assayed. Normalized data (*N* = 4), expressed as log_2_ (mRNA/GAPDH, usRNA/U6, or eRNA/U6), are plotted as means ± SE. *C*, A549 cells stably transfected with empty vector or reporter constructs for the (‒) orientation of P1+2, P1, P2, P3, and P4 GBSs were treated with siRNAs and Bud as in (*B*) prior to luciferase assay. The RLU measurements were normalized to that of the empty vector for each condition (siControl, siCBP, siP300, or siCBP + siP300). Normalized data (*N* = 4), expressed as log_2_ (RLU/RLU_empty_), are plotted as means ± SE. Significance, using normalized data relative to siControl group for each treatment condition was tested using ANOVA with Tukey’s post hoc test. ∗*p* ≤ 0.05, ∗∗*p* ≤ 0.01, ∗∗∗*p* ≤ 0.001.

To explore possible roles for P300 and CBP in the glucocorticoid-mediated induction of KLF9, siRNA-mediated knockdown of each was performed. This profoundly reduced expression of each factor without apparent off-target effects on the other (Fig. 7*A*). While P300 silencing modestly reduced KLF9 mRNA expression, neither CBP, nor P300, nor their combined silencing had any significant impact on either basal or budesonide-mediated induction of KLF9 mRNA or usRNA, and no changes in their fold induction were noted (Fig. 7*B*; Fig. S6*A*). Likewise, silencing of CBP, or P300, alone, had no significant effect on eRNA expression, whether in the absence or presence of budesonide. However, while CBP knockdown slightly reduced basal expression of some eRNAs, combined silencing of CBP plus P300 silencing reduced basal expression of all eRNAs (Fig. 7*B*). This was significant for eRNA 2.1 and 2.2 (Fig. 7*B*). However, as budesonide-induced expression of all four eRNAs was unaffected, fold induction of eRNAs 2.1 and 2.2 by budesonide was accordingly increased (Fig. S6*A*).

Next, the P1+P2(−) and P1−P4(−) reporter cells were subjected to siRNA knockdown of CBP, P300, or the combination prior to treatment with budesonide for 6 h. Knockdown of P300, or CBP, alone had little, or no, effect on either basal or budesonide-induced activity of the P1+2(−), P1(−), P2(−), or P4(−) reporters (Fig. 7*C*). However, basal and budesonide-induced activities of the P3(−) reporter were significantly increased by both CBP and P300 knockdown. Moreover, combined CBP/P300 knockdown further increased P3(−) activity in a manner that was more pronounced for the budesonide-treated group and led to significantly increased fold induction when compared with control (Fig. S6*B*). Conversely, combined CBP/P300 knockdown significantly decreased basal P2(−) reporter activity without a major effect on budesonide-induced activity, and this markedly increased the fold inducibility by budesonide (Fig. 7*C*, Fig. S6*C*). Furthermore, both basal and budesonide-induced activities of P1+P2(−), P1(−) and P4(−) were modestly, but often significantly, reduced by combined CBP/P300 knockdown (Fig. 7*C*). Collectively, these data suggest that CBP and P300 can modulate enhancer activity associated with GBSs upstream of the *KLF9* locus. However, despite this, neither factor appeared to be required for glucocorticoid-mediated induction of the endogenous *KLF9* gene in airway epithelial cells.

## Discussion

Regulation of gene expression by GR is not only central to numerous endocrine processes, but also critical for the control of inflammation by glucocorticoids administered for the treatment of inflammatory diseases, including asthma. Given their pleiotropic effects on many different target cells, it is perhaps not surprising that transcriptomic responses to glucocorticoids vary widely between different cell lines and cell types (10, 12, 56). Less explicable are the considerable differences observed between glucocorticoid-regulated transcriptomes in variants of epithelial cells in culture (8). Nevertheless, within the various data sets are genes that consistently show glucocorticoid responsiveness (8, 14, 57). This questions whether the mechanisms leading to these common responses are also conserved. To address this, the current study used *KLF9*, a gene that is robustly induced by glucocorticoids in primary HBE cells, two model epithelial cell lines, A549 and BEAS-2B (8), ASM cells, fibroblasts, endothelial cells, and macrophages (14, 33, 57). Furthermore, the additional finding that KLF9 mRNA and protein were induced by budesonide in HBE cells grown in ALI culture provides relevance to highly differentiated airway epithelium and is consistent with KLF9 being induced *in vivo* in the human airways post ICS inhalation (14).

KLF9 is one of 17 KLF transcription factors, many of which mediate effects of NHRs, including GR (41, 58, 59). While multiple NHRs may regulate KLF9 expression (36, 60, 61), in A549 cells THR and VDR ligands induced KLF9 to a lesser extent compared with glucocorticoids. Furthermore, KLF9 was the most robustly and consistently glucocorticoid-induced KLF in pulmonary epithelial cells, and this is mediated by GR, as confirmed by pharmacological and molecular approaches. Additionally, KLF9 induction was largely unaltered by the proinflammatory cytokine, IL1B, suggesting that interactions with core inflammatory pathways are not a major feature of KLF9 regulation.

Comparative analysis of A549 and BEAS-2B ChIP-seq data following dexamethasone treatment revealed four common glucocorticoid-induced GBSs located 5.9 (P1), 6.7 (P2), 25 (P3), and 65 (P4) kb upstream of the *KLF9* gene. These were confirmed by ChIP-PCR in A549 and primary HBE cells. Moreover, the P1 and P2 GBSs are reported in various murine cells, including adipocytes (62), neuronal cells (35), macrophages (33), and skeletal muscle cells (63). In fact, these sites are conserved in therian mammals, and the P1 site is conserved in tetrapods (35, 61). In addition, GRO-seq analysis of glucocorticoid-treated BEAS-2B cells showed robust induction of eRNAs from all GBSs. These data were confirmed by qPCR in A549 cells as well as in HBE cells grown in submersion and ALI culture. These effects were all blocked by GR silencing in A549 cells. Thus, glucocorticoid-mediated induction of KLF9 may represent an evolutionarily ancient process involving conserved GR-dependent transcriptional activation from multiple enhancers upstream of the *KLF9* gene.

Sequence examination of the four *KLF9* GBSs (P1–P4) showed a strong GRE motif at the center of each region and further supports a direct GR interaction with each GBS. Indeed, reporters containing each site plus flanking DNA exhibited GR-dependent induction of activity that was abolished in the P3 and P4 constructs by deletion of the GRE, thereby confirming a key role in transcriptional activation. Previous studies have shown loss of glucocorticoid-mediated induction of reporter activity from the P1 and P2 GBSs following point mutations to their GREs (35, 64, 65). Thus, intact GREs appear necessary for glucocorticoid-mediated induction of transcriptional activity from each *KLF9* GBS.

Consistent with the above observations, ChIP- and GRO-seq data in BEAS-2B cells revealed glucocorticoid-driven recruitment of Pol II and production of eRNA from the P4 and, to a lesser extent, the P3 GBSs. However, these data also revealed presence of Pol II, and production of nascent transcripts, at the P1/P2 region and around the *KLF9* TSS even in the absence of glucocorticoid. Constitutive transcriptional activity at the *KLF9* locus therefore appears to drive the basal expression of KLF9 mRNA and protein that was readily detectible by qPCR and western blotting, respectively. Notably, the P1/P2 region showed higher baseline eRNA production and more effectively drove constitutive reporter activity compared with the P3 and P4 regions. Such baseline activity was not inhibited by GR knockdown or blockade. Thus, even when isolated from its genomic context, the P1/P2 region recruits transcriptional machinery to activate transcription independently of GR binding. Indeed, numerous transcription factors, many of which confer constitutive transcriptional activity, may bind the P1/P2 region (Fig. S7) (66, 67). A key role for the P1/P2 region in driving basal KLF9 transcription is further supported by *in situ* Hi-C data (27), analysis of which suggests interaction between the *KLF9* P1/P2 and TSS regions in A549 cells even in the absence of glucocorticoid. Thus, constitutive transcriptional activity at the P1/P2 GBS region combined with interaction with the TSS region may drive basal KLF9 expression.

**Figure 8:**
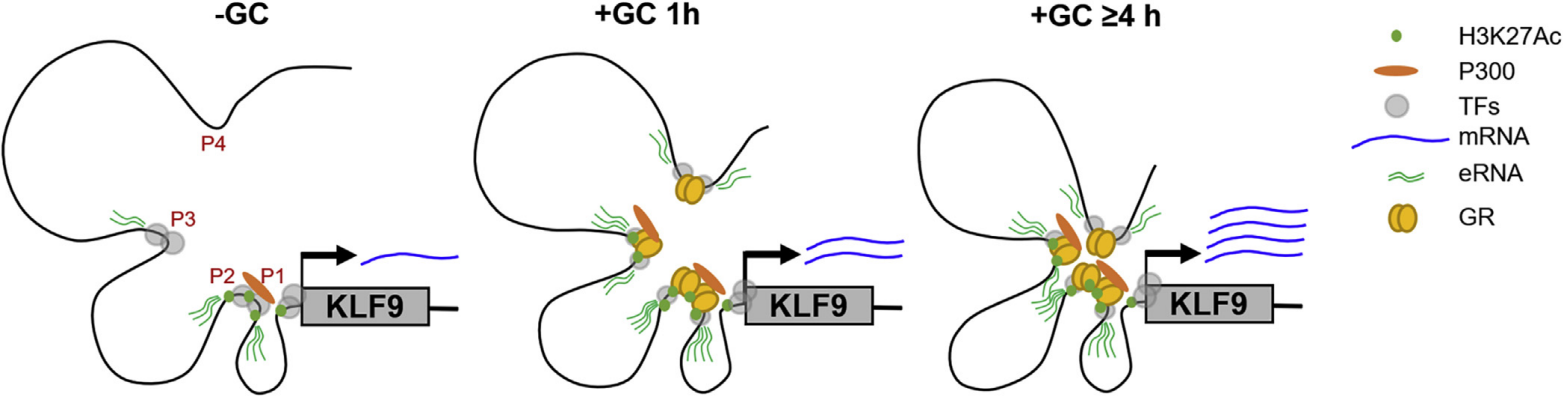
**A model for glucocorticoid regulation of KLF9.** Schematic representation of genomic events that control KLF9 expression in the absence or presence of glucocorticoids (GCs). In the absence of glucocorticoids, basal KLF9 expression is driven *via* constitutive transcriptional activity from the proximal P1/P2 region and its interaction with the TSS (*left*). Upon glucocorticoid exposure, GR is rapidly recruited to all 4 KLF9 GBSs. This promotes local production of eRNAs and increases interaction of distal enhancers, P3 and P4, with P1/P2 and the TSS. These events rapidly increase KLF9 transcription leading to increased KLF9 expression (*center*). With the progression of time, increased interactions between these regions enable greater levels of KLF9 transcription (*right*).

Analysis of Hi-C data in A549 cells revealed that glucocorticoid treatment and GR recruitment to upstream *KLF9* GBSs led to marked changes in the chromatin landscape of this region. While interaction between the TSS and P1/P2 region was largely unaffected by glucocorticoid, interactions between the P3 and P4 GBSs and TSS and/or P1/P2 region were enhanced in a time-dependent manner. These effects, while correlating with the induction of KLF9 mRNA and usRNA, which started low and increased until peaking around 6 h, contrast the induction of eRNAs that was more constant over time. The above observations suggest that immediate activation of the P1/P2 enhancers, which constitutively interact with the *KLF9* TSS region, combined with rapid onset interactions involving P3 and P4, may trigger initial rapid transcriptional activity. As long-range chromatin interactions consolidate, the distal GR-activated enhancers, P3 and P4, could contribute further toward increased KLF9 transcription. Indeed, eRNAs produced from these GBSs may contribute to consolidating chromatin loops by aiding recruitment and positioning of the mediator complex (68, 69), an effect that could also explain baseline interactions between the TSS and the transcriptionally active P1/P2 region. Collectively, these data suggest a model whereby basal KLF9 expression is driven *via* constitutive transcriptional activity from the proximal P1/P2 region and its interaction with the TSS (Fig. 8). Upon glucocorticoid exposure, GR is rapidly recruited to all four KLF9 GBSs. This promotes the local production of eRNAs and increases interaction of the distal enhancers, P3 and P4, with P1/P2 and the TSS. These events rapidly increase KLF9 transcription leading to increased KLF9 expression. With the progression of time, increased interactions between these regions enable greater levels of KLF9 transcription (Fig. 8).

Histone modifications, such as H3K27Ac, along with the presence of histone acetyltransferases, including CBP and P300, mark active enhancers and are important for activation by sequence-specific transcription factors (69, 70, 71). Thus, H3K27Ac enrichment at the *KLF9* P1/P2 region in A549 cells and the presence of P300, as apparent from the data of McDowell and coworkers (24), are consistent with constitutive transcriptional activity at this region. Furthermore, the time-dependent enrichment of P300 at the P1/P2 and P3 GBSs following glucocorticoid treatment points to a role for P300/CBP in KLF9 transcriptional regulation. However, robust silencing of CBP, P300, or both, produced no detectable change in the glucocorticoid-mediated induction of KLF9 mRNA or usRNA. Despite this, eRNA production and reporter activity from the P1, P2 and P4 GBSs were reduced, most notably with respect to basal activity of the P2 region. These data are thus more consistent with a role for CBP/P300 in maintaining basal activity from these enhancers, in particular P2. However, the lack of effect on KLF9 mRNA or usRNA is more challenging to interpret. Coactivator recruitment and effects on eRNA production could be redundant, at least at the 6 h time point. Alternatively, a convoluted regulatory framework could exist with CBP and/or P300 modulating competing aspects of KLF9 regulation. In this regard, double knockout of CBP/P300 has been associated with both negative and positive impacts on gene expression (72). Accordingly, we observed significantly increased P3 GBS reporter activity following silencing of CBP, P300, or both. We therefore speculate on the existence of a more complex regulatory environment for the control of KLF9 expression that involves both positive and negative effects of coactivators, such as CBP and P300, mediated in a combinatorial fashion through different GBSs. This intriguing possibility is further hinted at by the finding that H3K27Ac was high at baseline in the P1/P2 region and decreased with glucoocrticoid treatment, while the P3 and, to a lesser extent, the P4 region showed low basal H3K27Ac that increased with glucocorticoid treatment.

In summary, glucocorticoids upregulate KLF9 expression in numerous cell types, including cells relevant to the airways. This occurs in human pulmonary epithelial cell lines, undifferentiated primary HBE cells grown in submersion culture, and highly differentiated HBE cells grown at ALI. Demonstration that glucocorticoids induce not only KLF9 gene expression, but also binding of GR to multiple conserved regions in cell line models and primary cells, supports the rationale for using cell lines to interrogate mechanisms of gene regulation. That transcriptional activity from these GBSs occurs in highly differentiated HBE cells grown at ALI implies similar mechanisms *in vivo*, for example, with ICS therapy. Mechanistically, these data indicate a proximal enhancer region that contains two GBSs (P1 and P2) as interacting with the *KLF9* TSS region and driving basal KLF9 expression (Fig. 8). Following glucocorticoid exposure, transcriptional activity at these sites, plus more distal GBSs (P3, P4), rapidly increases along with KLF9 gene transcription. Interaction between these regions and the KLF9 TSS increases with time and may contribute to time-dependent increases in KLF9 transcription. While the coactivator, P300, was enriched at the P1/P2 region under basal conditions and this increased with glucocorticoid treatment, no clear role for P300 and/or CBP in the regulation of KLF9 was established. Taken together, these data suggest a model for regulation of gene expression by glucocorticoid that is highly conserved in multiple systems and provides an unequivocal demonstration that mechanistic insight gained from cell line models can translate to physiologically relevant systems.

## Experimental procedures

### Drugs

Budesonide (gift from AstraZeneca, Sweden), Org34517 (gift from Chiesi Farmaceutici, Parma, Italy), progesterone (57-83-0, Cayman Chemical), 17β-estradiol (50-28-2, Cayman Chemical), dexamethasone (D8893), triiodo-l-thyronine (T2877), retinoic acid (R2625), 9-*cis*-retinoic acid (R4643), 7β-hydroxycholesterol (H6891), and hemin (51280) (all from Sigma-Aldrich) were dissolved in dimethyl sulphoxide (DMSO) (D2650, Sigma-Aldrich) as stocks of 1 to 10 mM. Final DMSO concentrations on cells were ≤0.1%. Dihydroxy vitamin D3 (D1530, Sigma-Aldrich) was dissolved in absolute ethanol as a stock of 10 mM. Recombinant human IL1B (201-LB, R&D Systems) was dissolved in phosphate-buffered saline (PBS) (14190144, Thermo) containing 0.1% bovine serum albumin (BSA) (A3059, Sigma-Aldrich). G418 (A1720, Sigma-Aldrich) was dissolved in sterile water as stocks of 100 mg/ml.

### Submersion cell culture

The human pulmonary type II cell line, A549 (American Type Culture Collection; ATCC), was grown in Dulbecco’s modified Eagle’s medium (DMEM) (11995065, Thermo) supplemented with 10% fetal bovine serum (FBS) (A3160702, Thermo) and 2 mM L-glutamine (25030081, Thermo). HBE BEAS-2B cells (ATCC) were grown in DMEM/F12 (11330057, Thermo) supplemented with 14 mM NaHCO_3_, 2 mM L-glutamine, and 10% FBS. Primary HBE cells were isolated from non transplanted normal human lungs obtained through a tissue retrieval service at the International Institute for the Advancement of Medicine (Edison, NJ), as previously described (73, 74). No personal identifying information was provided for any of the donors, and local ethics approval for the use of human tissues was granted by the Conjoint Health Research Ethics Board of the University of Calgary. HBE cells were grown in submersion culture in bronchial epithelial cell growth medium (BEGM) (CC-3171, Lonza) containing SingleQuots supplements (CC-4175, Lonza). All cells were incubated at 37 °C in 5% CO_2_ and were grown to confluence either in 6 or in 12-well plates, as appropriate. Prior to experiments, cells were incubated overnight in basal media that was serum- and additive-free.

### Air–liquid interface culture of HBE cells

ALI culture of primary HBE cells was performed as previously described (75). HBE cells were seeded into T-75 cm^2^ flasks in PneumaCult-EX expansion medium (05009, StemCell) containing 50X Supplement (05019, StemCell), 25 μg/ml fluconazole (F8929, Sigma-Aldrich), 100 U/ml penicillin/streptomycin (15140-122, Thermo), and 1 μM hydrocortisone (07904, StemCell). At 90% confluence, cells were lifted using Trypsin/EGTA (CC-5012, Lonza) and Trypsin Neutralizing Solution (CC-5002, Lonza), seeded at a density of 2.0 × 10^5^ cells/cm^2^ into transwell inserts (3408, Corning) coated with bovine collagen Type I/III (5005-B, Advanced BioMatrix) and maintained at 37 °C, 5% CO_2_. Forty-eight hours postseeding, the apical media was removed to expose the cells to air and fed basally with PneumaCult-ALI Differentiation Medium (05002, StemCell) containing 100X Supplement (05003, StemCell), 25 μg/ml fluconazole, 100 U/ml penicillin/streptomycin, 1 μM hydrocortisone, and 4 μg/ml heparin (07980, StemCell). Cells were fed basally every 48 h. From 14 days post transwell seeding, cells were washed apically once per week with DPBS to remove accumulated mucus from goblet cell differentiation. Highly differentiated ALI cultures were used for experiments 5 weeks post transwell seeding. For experiments, ALI cultures were fed basally with PneumaCult-ALI Basal Media (no supplements) 18 h prior to experiments. Prior to drug exposure, cells were washed with DPBS to remove excess mucus and fresh medium containing drugs and stimuli were added both apically and basolaterally to maximize exposure. Apical treatments were diluted in 0.025 M HEPES in F12 (200 μl/well), and basolateral treatments were diluted in PneumaCult-ALI Basal Media (1 ml/well).

### Western blot analysis

Cells were lysed in RIPA buffer (9806, Cell Signaling) and then mixed 1:3 with 4x Laemmli sample buffer (4% SDS, 10% 2-mercaptoehtanol, 20% glycerol, 0.004% bromophenol blue, 0.125 M Tris HCl, pH 6.8.), followed by boiling for 10 min at 100 °C before loading onto gels. Size-fractionated proteins were transferred to nitrocellulose membranes prior to blocking and incubated with KLF9 (sc-376422, SantCruz), GR (PA1-511A, Thermo), CBP (7389, Cell Signaling), P300 (86377, Cell Signaling), or GAPDH (MCA4739, Bio-Rad) overnight at 4 °C according to the manufacturer’s recommendations. Membranes were then washed and incubated with horseradish-peroxidase-linked secondary immunoglobulin (Jackson ImmunoResearch) for 1 h at room temperature. Immune complexes were detected by enhanced chemiluminescence (Bio-Rad). Images were acquired using a ChemiDoc Touch imaging system (Bio-Rad) prior to analysis using ImageLab software (Bio-Rad). Band volumes for the protein of interest were normalized to GAPDH.

### RNA isolation, cDNA synthesis, and SYBR green real-time PCR

Total RNA was extracted using the NucleoSpin RNA Extraction kit (MN-740955, Macherey-Nagel) and cDNA prepared from 0.5 to 1 μg of RNA. After being diluted 1:4, PCR was carried out on 2.5 μl of cDNA using Fast SYBR Green Master Mix (4385618, Thermo) with a QuantStudio3 PCR system (Thermo). Relative cDNA concentrations were obtained from standard curves generated by serial dilution of cDNA obtained from RNA of glucocorticoid-treated samples and analyzed at the same time as experimental samples. Amplification conditions were: 95 °C for 20 s, then 40 cycles of 95 °C for 3 s, 60 °C for 30 s. Primer pairs specific to regions/genes of interest are listed in Table S1. All primers were designed using PrimerBLAST (NCBI) and were synthesized by the DNA synthesis lab at the University of Calgary. Primer specificity was determined using dissociation (melt) curve analysis: 95 °C for 3 s, 60 °C for 30 s followed by ramping to 95 °C at 0.1 °C/s with continuous fluorescence measurement. A single peak in the change of fluorescence with temperature indicates acceptable specificity of primers. The quantity of the target from two technical replicates was averaged and normalized to the mean quantity of GAPDH (for mRNA) or U6 (for usRNA and eRNA) determined from the same cDNA sample.

### Chromatin immunoprecipitation

A549 or HBE cells in 100-mm or 6-well cell culture plates, respectively, grown to >80% confluence were serum/additive starved overnight before treatments. Protein-DNA cross-linking was performed by adding 16% methanol-free formaldehyde (PI28906, Thermo) directly to the culture medium to a final concentration of 1% and incubating for 10 min at room temperature. Formaldehyde was then quenched at room temperature for 5 min with 125 mM glycine. The cells were then washed for 5 min with ice-cold PBS prior to scraping into ice-cold cell lysis buffer (5 mM PIPES pH 8.0, 1 mM EDTA, 85 mM KCl, 5% glycerol, 0.5% NP-40) supplemented with protease inhibitor cocktail (PIC) (PI-78439, Thermo). The cell suspension was incubated at 4 °C for 2 h with continuous agitation. Nuclei were collected by centrifugation (600*g*, 5 min, 4 °C) and resuspended in ice-cold nuclei lysis buffer (NLB) (1× PBS containing 1 mM EDTA, 5% glycerol, 0.5% sodium deoxycholate, 0.1% SDS, and 1% NP-40) supplemented with PIC. Samples were sonicated at 4 °C using a Bioruptor (Diagenode) and 28 to 30 high-power bursts with a 30 s on-off cycle. Lysates were cleared by centrifugation (maximum speed for 15 min at 4 °C) and supernatants were used for immunoprecipitation. Protein G magnetic Dynabeads (10004D, Thermo) were preincubated with 10 μg GR-356 antibody (20) overnight at 4 °C in NLB supplemented with PIC and 5 mg/ml BSA. After washing twice with ice-cold NLB + PIC, the Dynabeads were incubated with cleared lysates overnight at 4 °C in NLB supplemented with PIC and BSA. Beads were then subjected to four washes with ice-cold NLB containing 500 mM NaCl, followed by four washes with ice-cold LiCl buffer (20 mM Tris at pH 8.0, 1 mM EDTA, 250 mM LiCl, 0.5% NP-40, 0.5% sodium deoxycholate). Cross-links were reversed by incubating the beads in TE buffer (10 mM Tris-HCl + 1 mM EDTA, pH 8.0) supplemented with 0.7% SDS and 0.2 mg/ml Proteinase K (P6556, Sigma-Aldrich) for 3 h at 55 °C, then 16 h at 65 °C. DNA was then purified with a ChIP DNA Clean & Concentrator kit (D5205, Zymo Research) prior to qPCR using Fast SYBR Green Master Mix (Thermo) as described above. ChIP-PCR primers were designed to span a GR binding site (GBS) in an *FKBP5* intronic region (as positive control) as well as the four GBSs upstream of the *KLF9* gene, as identified in the GR ChIP-seq data of dexamethasone-treated BEAS-2B and A549 cells (19, 24). ChIP-PCR data were normalized to the geometric mean of three negative control regions, *OLIG3*, *MYOD1*, and *MYOG*, not predicted to be occupied with GR (44, 45, 76). Primer pairs specific to regions of interest are listed in Table S2.

### siRNA-mediated gene silencing

A549 cells at 60 to 70% confluence in 12-well plates were transfected with siRNAs. Pools of four nontargeting siRNAs (SI03650325, SI03650318, SI04380467, 1022064), GR siRNA (SI00003745, SI00003766, SI02654757, SI02654764), CBP siRNA (SI02633099, SI02633092, SI02633085, SI02622648), or P300 siRNA (SI03078761, SI03038259, SI02626267, SI02622592) (all from Qiagen) were mixed with 3 μl of Lipofectamine RNAiMax (13778150, Thermo) in 100 μl of Opti-MEM (31985070, Thermo) and then incubated at room temperature for 5 min. Mixtures were then incubated on cells in the presence of serum for 24 to 48 h; final siRNA concentrations on cells were 1 nM. Prior to experiments, cells were incubated overnight in serum- and additive-free basal medium.

### Plasmids, transfection, and luciferase assay.

PCR primers were designed to amplify each KLF9 GBS, with its flanking DNA (300–700 bp), as well as a larger region harboring the P1 and P2 sites (P1+2; 1300 bp) (Table S3). PCR fragments were amplified using Platinum SuperFi PCR Master Mix (12358010, Thermo) and then introduced into linearized and topoisomerase I-activated pCR Blunt II-TOPO vector (450245, Thermo). Both orientations, (+) and (‒) strand, of each region were subcloned into pGL3.TATA.neo vector (77) using *KpnI* (R3142, New England Biolabs) and *XhoI* (R0146, New England Biolabs) enzymes. Mutagenesis of the GRE sequences of the P3 and P4 GBSs was performed using the Q5 Site-Directed Mutagenesis Kit (E0554S, New England Biolabs) as suggested by the manufacturer. Primer pairs used for mutagenesis are listed in Table S4. Each of these plasmids and the empty vector (as control) were transfected into A549 cells using Lipofectamine 2000 (11668019, Thermo) 24 h before the addition of 1 mg/ml G418. After 14 to 21 days, G418-resistant cells were passaged in the presence of G418 (0.6 mg/ml), then frozen down, and stored in liquid nitrogen for future experiments. For reporter experiments, stably transfected A549 cells were plated in 24-well plates and incubated until confluent in the presence of G418 (0.6 mg/ml). For siRNA knockdown experiments, stably transfected A549 cells were plated in the absence of G418. Prior to treatments, cells were incubated overnight in serum- and additive-free basal medium. Reporter assays were performed using the Firefly Luciferase Assay Kit 2.0 (30085, Biotium Inc) according to manufacturer’s protocol.

### Data analysis and graphical presentation

Significant changes in normalized expression, fold change, or reporter activity were identified by ANOVA followed by Tukey’s Honest Significant Difference post-hoc test (for comparing three or more groups) or Student’s *t*-test (for comparing 2 groups). Data figures were generated using R packages: “*pheatmap*” was used to produce heatmaps, “*drc*” was used for concentration response curve generation, and “*ggplot2*” was used to produce all other figures. Data are summarized as line graphs depicting means ± standard error (SE) or as box-and-whiskers plots, where whiskers represent min and max values and boxes represent lower and upper quartiles and median values. Genomic regions as well as ChIP-seq and GRO-seq data were visualized using the UCSC Genome Browser (https://genome.ucsc.edu). To maintain consistency between figures, the default GRCh38 convention for positive (+) and negative (‒) strand orientation was adopted, where the *KLF9* gene is shown as a (‒) strand gene. Hi–C interaction matrices were generated by the “Juicebox” webtool (https://aidenlab.org/juicebox; (51)). GRE motifs within KLF9 GBSs were identified using JASPAR CORE 2020 track in UCSC Genome Browser (49).

## Data availability

All data produced for this work are contained within the article and the supporting information.

## Conflict of interest

The authors declare that they have no conflicts of interest with the contents of this article.
