# Supplementary material for: Genomic determinants implicated in the glucocorticoid-mediated induction of KLF9 in pulmonary epithelial cells
Source: J Biol Chem. 2020 Nov 23;296:100065. doi: 10.1074/jbc.RA120.015755 (PMC7949084; doi:10.1074/jbc.RA120.015755)
Supplement: Supplementary Figures and Tables [file mmc1.pdf]

Supporting Information

Genomic determinants implicated in the glucocorticoid-mediated induction of KLF9 in pulmonary epithelial cells

Mahmoud M. Mostafa[1], Akanksha Bansal[1], Aubrey N. Michi[1], Sarah K. Sasse[2], David Proud[1], Anthony N. Gerber[2, 3], Robert Newton[1]*

[1]Department of Physiology & Pharmacology and Snyder Institute for Chronic Diseases, Cumming School of Medicine, University of Calgary, 3330 Hospital Dr NW, Calgary, AB T2N 4N1, Canada

[2]Department of Medicine, National Jewish Health, Denver, Colorado 80206, USA

[3]Department of Medicine, University of Colorado, Aurora, Colorado 80045, USA

*Corresponding author: Robert Newton; rnewton@ucalgary.ca

Contents

Table S1. Sequences of primers used for qPCR analysis of cDNA
Table S2. Sequences of primers used for qPCR analysis of ChIP DNA
Table S3. Sequences of primers used for cloning of KLF9 GBSs
Table S4. Sequences of primers used for site-directed mutagenesis of KLF9 P3 and P4 GBSs
Figure S1. Regulation of KLF9 expression in the airway cells.
Figure S2. GR binding to the genomic region around the KLF9 gene.
Figure S3. Baseline transcriptional activity at KLF9 GBSs.
Figure S4. GRE motifs at the center of GBSs upstream of KLF9.
Figure S5. H3K27Ac marks near *KLF9* gene.
Figure S6. Effect of CBP and P300 knockdown on the glucocorticoid-mediated induction of KLF9 expression and enhancer activity from KLF9 GBSs.
Figure S7. Transcription factor binding to the P1/P2 GBS.

Table S1. Sequences of primers used for qPCR analysis of cDNA samples

Target	Forward Primer (5′-3′)	Reverse Primer (5′-3′)
**GAPDH**	TTCACCACCATGGAGAAGGC	AGGAGGCATTGCTGATGATCT
**KLF9 mRNA**	CCTCCCATCTCAAAGCCCATT	TCGTCTGAGCGGGAGAACTT
**KLF9 usRNA**	GCCTGGCCAAAGGTTAACTA	TCCCATCTCAAAGCCCATTAC
**KLF9 eRNA 1**	CCTGGGTGTTAGCTGTCATTA	GGGAGAGGCTAAAGGGATAAAG
**KLF9 eRNA 2.1**	GGTTGGGAAAGTGCAGGAT	GTAGGTGTAGGGAGGTGGATAG
**KLF9 eRNA 2.2**	CCTGCCTGGCTGTCTTT	GTTAGCTGTCTCACCACTTCTC
**KLF9 eRNA 4**	GTTTACACAGGGCAGCAAAC	GATGGCTGGTCACAATCATAGA
**U6**	AATTGGAACGATACAGAGAAGATTAGC	GGAACGCTTCACGAATTTGC

Table S2. Sequences of primers used for qPCR analysis of ChIP DNA samples

Target	Forward Primer (5′-3′)	Reverse Primer (5′-3′)
**FKBP5**	TAACCACATCAAGCAAGCTG	GCATGGTTTAGGGGTTCTTG
**KLF9 P1**	CCCAATCTAGGGCAGTTTGTT	TGAGGAGGCGAGTTCATCT
**KLF9 P2**	TGAGCGCTATATTTACCAAGGG	GAGTCAGAATGGGCTGTGTT
**KLF9 P3**	CTCACCTCAGGAACTTTGTACTT	AGCTAAGCTGAGGAGGAGTTA
**KLF9 P4**	CGAGAGATCACCACGATTTCA	GAGGAACCACACAGACAAAGA
**MYOD1**	TGCAGGAGATGAAATACTAAGCAAGTA	AGATTGGAAACTGAGGACTTTAGTTAGAG
**MYOG**	CCAATGAGACTGAGTGGGTTTTC	TCACCAGAGAAGACTGCTTTGC
**OLIG3**	GGCAAGGACAGAGACAATCATA	CTCTGTGTTCTCGCTTTGGA

Table S3. Sequences of primers used for cloning KLF9 GBSs

DNA region	Forward Primer (5′-3′)	Reverse Primer (5′-3′)	Size (bp)
**KLF9 P1+2**	GCGAGGTTATGCGAGGTAA	GGAGGATAAGTTTAGCTCGCA	1289
**KLF9 P1**	TGTCATCTCGACACCCTACT	GGAGGATAAGTTTAGCTCGCA	679
**KLF9 P2**	GCGAGGTTATGCGAGGTAA	GTTAGGAAACATTCCTGCCATTC	409
**KLF9 P3**	TGCTGTTGCCCTTGGATAAA	GGCTTGGCACTGTGGATATAA	430
**KLF9 P4**	GTCCAGAGATCGTCTTTGACAG	TCAGGAAGTGTAATGCTTGGG	339

Table S4. Sequences of primers used for site-directed mutagenesis of KLF9 P3 and P4 GBSs

GBS	Forward Primer (5′-3′)	Reverse Primer (5′-3′)
**ΔGRE.KLF9 P3**	ACAAAGTTCCTGAGGTGAG	TCCCTCTTCCTGGAACATC
**ΔGRE.KLF9 P4**	ACTGGCACAACAGGCTGG	TCTCTTCTGTGAACTGAGTTTGAAC

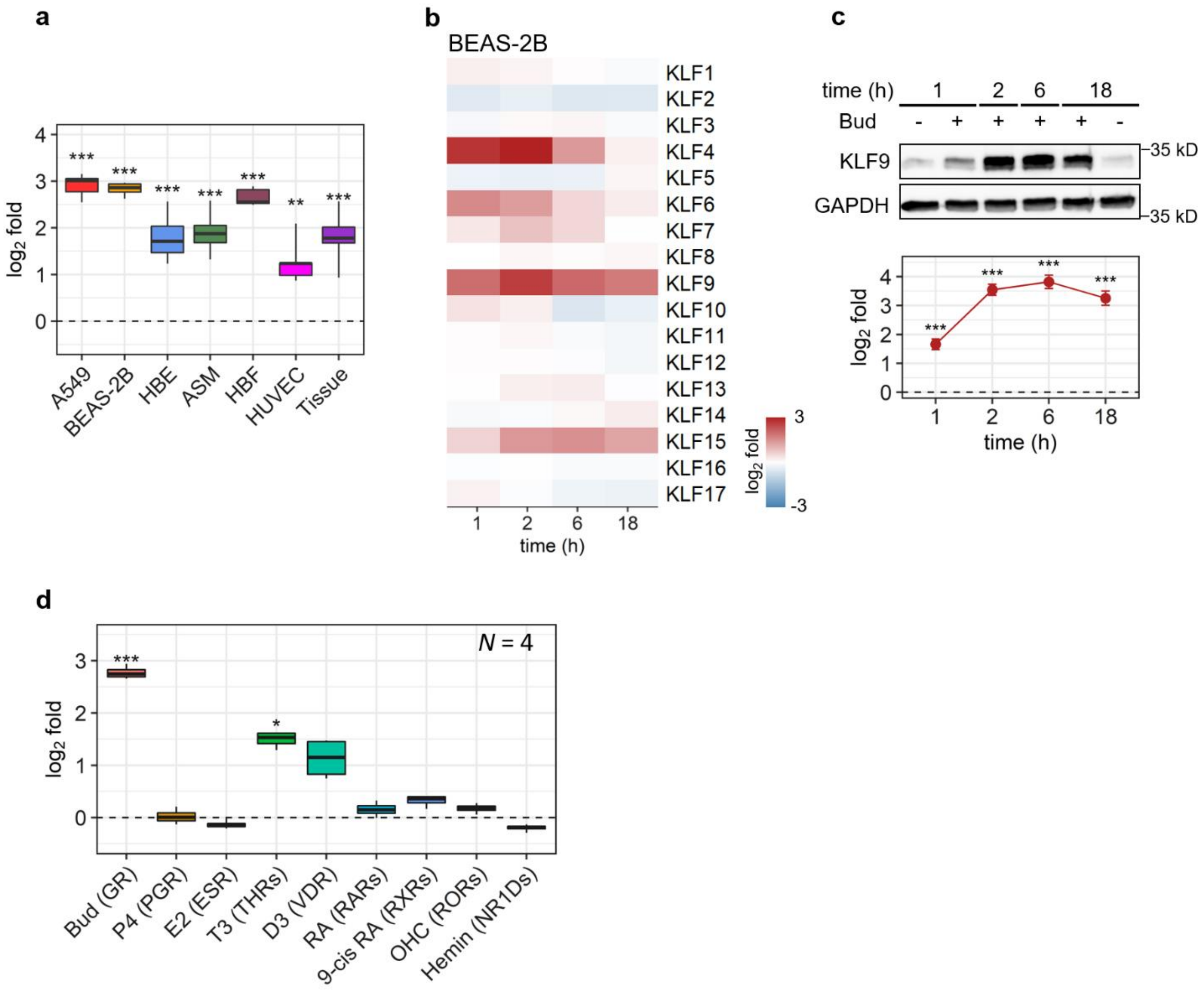

Figure S1. Regulation of KLF9 expression in the airway cells. a, Compilation of previously reported qPCR data showing budesonide-mediated induction of KLF9 in A549 and BEAS-2B cell-lines, human bronchial epithelial (HBE), airway smooth muscle (ASM), bronchial fibroblast (HBF) and vascular endothelial (HUVEC) primary cells, as well as airway tissue (8, 14). Data (*N* = 6-11), expressed as KLF9/GAPDH, were plotted as $\log_2$ fold change relative to control in each dataset. Data are plotted as box-and-whiskers plots. Significance, using normalized KLF9/GAPDH values to no treatment control was tested by paired *t* test. **b**, Microarray analysis (*N* = 4) of BEAS-2B cells treated with 300 nM budesonide for the indicated times. The heatmap depicts the effect ($\log_2$ fold) of budesonide on the expression of the 17 KLFs when compared to no treatment control at each time point. **d**, BEAS-2B cells were either not treated or treated with 300 nM budesonide (Bud) for the indicated times prior to western blot analysis of KLF9 and GAPDH. Representative blots are shown (upper panel). Following densitometric analysis, data (*N* = 4), as KLF9/GAPDH were expressed as $\log_2$ fold relative to no treatment at 1 h and are plotted as means ± SE (lower panel). Significance, using normalized KLF9/GAPDH values relative to control at 1 h, was tested by ANOVA with Tukey's post-hoc test. **e**, A549 cells were either not stimulated or treated with 300 nM budesonide (Bud), 1 µM progesterone (P4), 1 µM estradiol (E2), 1 µM triiodo-L-thyronine (T3), 100 nM dihydroxyvitamin D3 (D3), 1 µM retinoic acid (RA), 1 µM 9-cis-retinoic acid (9-cis RA), 10 µM 7β-hydroxycholesterol (OHC), or 1 µM hemin, as an agonists for the glucocorticoid (GR), progesterone (PGR), estrogen (ESR), thyroxine (THRs), vitamin D (VDR), retinoic acid (RARs), retinoid (RXRs), RAR-related (RORs), or Rev-Erb (NR1Ds) receptors,

respectively. Cells were harvested for RNA 6 h after treatment and qPCR was performed for KLF9 and GAPDH. Data (N = 4), expressed as KLF9/GAPDH, were plotted as $\log_2$ fold change relative to no treatment control. Significance, using normalized KLF9/GAPDH values relative to no treatment control was tested by ANOVA with Tukey's post hoc test. * $P \leq 0.05$, ** $P \leq 0.01$, *** $P \leq 0.001$.

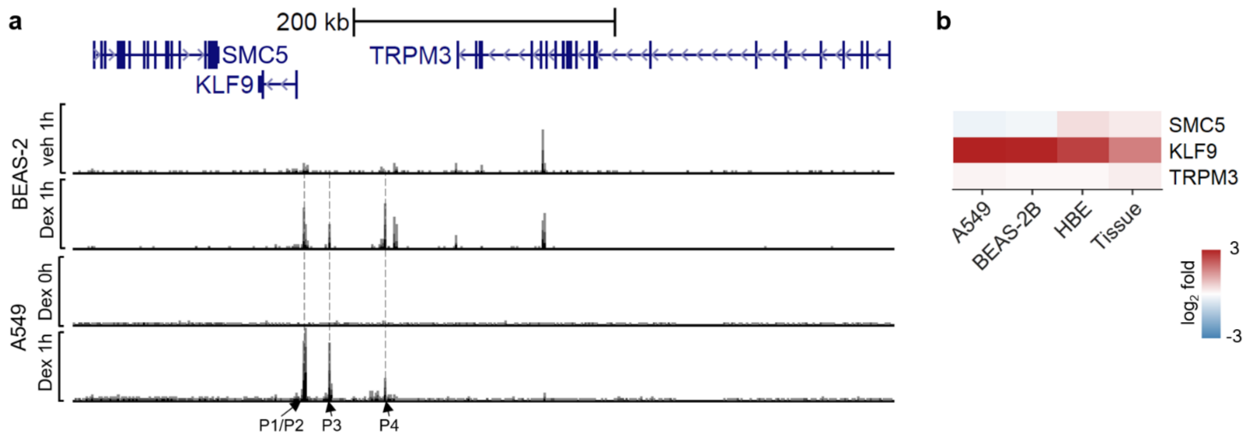

Figure S2. GR binding to the genomic region around the *KLF9* gene. a, Genome browser snapshot of the *KLF9* locus showing the nearby genes, *SMC5* and *TRPM3*. Arrow heads within the intronic regions indicate direction of transcription. GR ChIP-seq traces following 1 h treatment in BEAS-2B cells with vehicle control (veh) or 100 nM dexamethasone (Dex) are shown (data from Kadiyala *et al.*, 2016 (19); upper two tracks). GR ChIP-seq traces in A549 cells treated with 100 nM Dex for the indicated times are also displayed (data from McDowell *et al.*, 2018 (24); lower two tracks). Dashed lines indicate the position of GR binding sites (P1-4) characterized in figure 3. **b**, Microarray analysis performed on A549, BEAS-2B, primary human bronchial epithelial (HBE) (8), and human bronchial biopsies (tissue) (14). Cells were exposed to maximally effective concentrations of budesonide (300 nM for A549 and BEAS-2B, or 100 nM for HBE) for 6 h prior to harvest. Bronchial biopsies were collected ~6 h post high-dose budesonide (1600 µg) inhalation. Heatmap depicts the effect (log_2 fold) of budesonide treatment on the mRNA expression of KLF9, SMC5 and TRPM3, as compared to time-matched no treatment control, for cultured cells, or placebo inhalation, for the tissues.

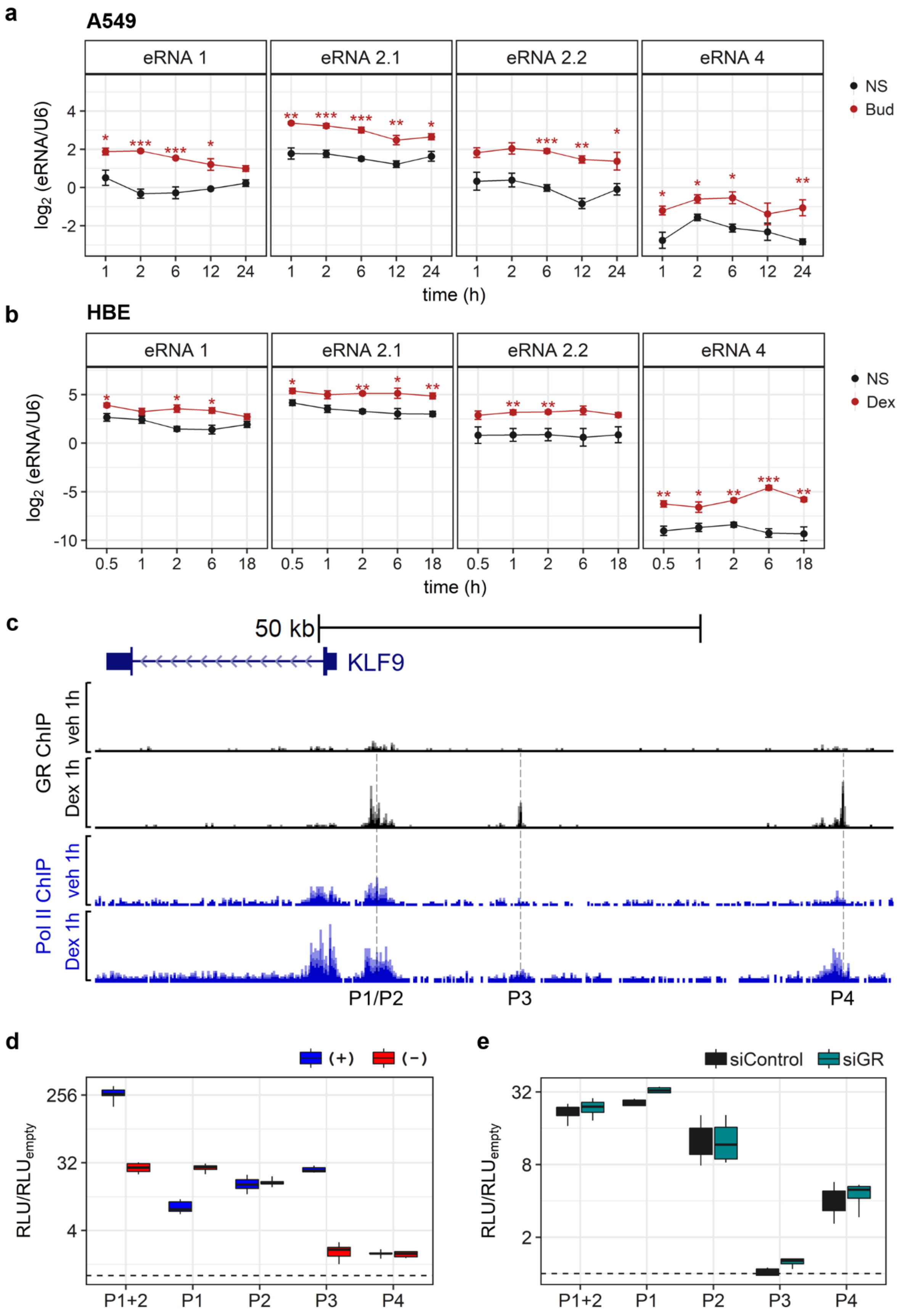
a
A549
eRNA 1
eRNA 2.1
eRNA 2.2
eRNA 4
NS
Bud
log2 (eRNA/U6)
time (h)
b
HBE
eRNA 1
eRNA 2.1
eRNA 2.2
eRNA 4
NS
Dex
log2 (eRNA/U6)
time (h)
c
50 kb
KLF9
GR ChIP
veh 1h
Dex 1h
Pol II ChIP
veh 1h
Dex 1h
P1/P2
P3
P4
d
(+)
(−)
RLU/RLUempty
P1+2
P1
P2
P3
P4
e
siControl
siGR
RLU/RLUempty
P1+2
P1
P2
P3
P4

Figure S3. Baseline transcriptional activity at KLF9 GBSs. A549 (**a**) or primary HBE (**b**) cells were either not stimulated (NS) or treated with 300 nM budesonide (Bud; **a**) or 1 µM dexamethasone (Dex; **b**) for the indicated times before cells were harvested for RNA and qPCR was performed for U6 and the four eRNAs, 1, 2.1, 2.2, and 4. Normalized data (N = 4), as $\log_2$ (eRNA/U6), are plotted as means ± SE. Significance, using normalized eRNA/U6 values relative to NS at each time point was tested by paired *t* test. **c**, Genome browser snapshot of *KLF9* gene along with ~70 kb of the 5′ upstream region showing ChIP-seq traces for GR (black) and Pol II (blue) in BEAS-2B cells following treatment with vehicle control (veh) or 100 nM Dex for 1 h (data from Kadiyala *et al.*, 2016 (19)). Dashed lines indicate the position of GR binding sites (P1-4) identified in figure 3. **d**, A549 cells stably transfected with empty vector or luciferase reporter constructs for each of the GBSs upstream to *KLF9* were harvested for reporter assay. Normalized data (N = 4), expressed as RLU/RLU_{empty}, are plotted as box-and-whiskers plots. Reporter activity from GBSs with (+) or (–) orientation are shown in blue or red, respectively. **e**, A549 cells stably transfected with empty vector or reporter constructs for P1+P2(–) and P1 – P4(–) GBSs were incubated with either 1 nM of a pool of 4 non-targeting siRNAs (siControl; black) or 1 nM of a pool of 4 siRNAs targeting GR (siGR; green) for 48 hours. Cells were harvested for reporter assay. Normalized data (N = 4), expressed as RLU/RLU_{empty}, are plotted as box-and-whiskers plots.

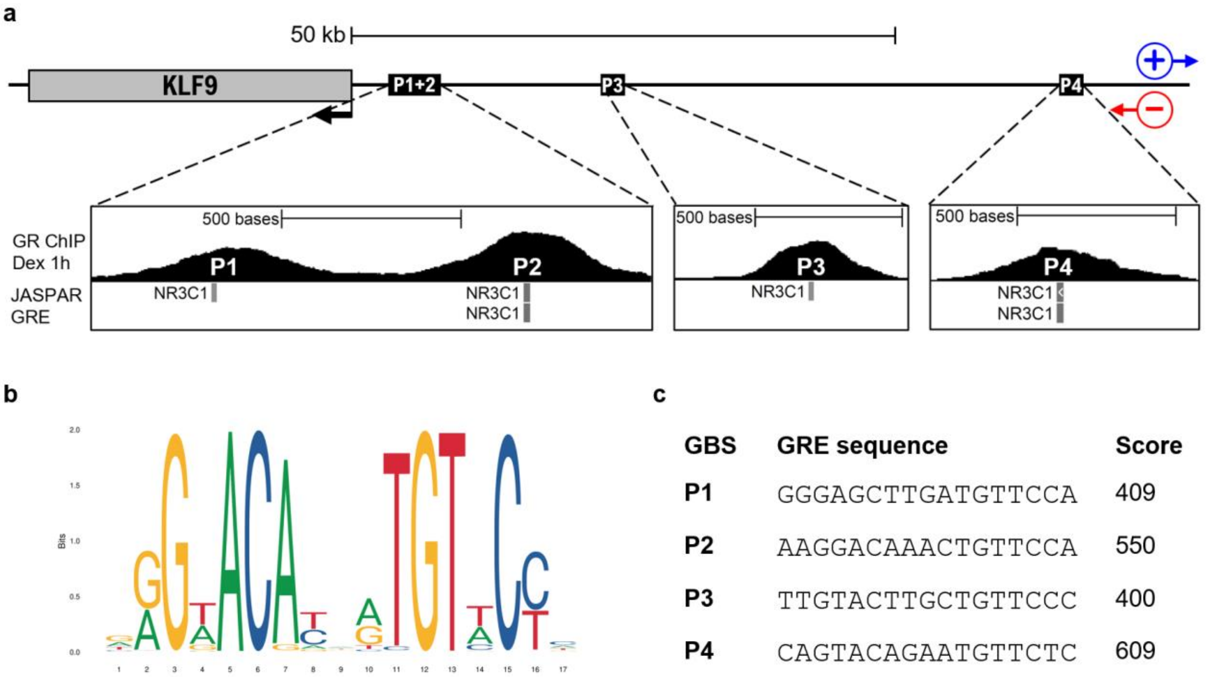

Figure S4. GRE motifs at the center of GBSs upstream of *KLF9*. **a**, Schematic of the *KLF9* gene along with ~70 kb of the 5′ upstream region harboring the GBSs (black boxes) described in figure 3. Insets show zoomed-in genome browser snapshots for GR ChIP-seq traces of the corresponding region in A549 cells treated with 100 nM Dex for 1 h (data from McDowell *et al.*, 2018 (24)). The position of GRE sequence, as predicted by JASPAR CORE database for GR (NR3C1) binding sites (score ≥ 400), is highlighted below each GR ChIP peak. **b**, Position weight matrix (PWM) of GR binding motifs in human cells, as identified by JASPAR CORE database (49). **c**, The sequence of the GREs identified within each GBS is shown with their corresponding score. Scores are calculated based on the probability of candidate sequence to match GR PWM (**b**), where 0 corresponds to a P value of 1 and 1000 to a P value ≤ 10^{-10} (50).

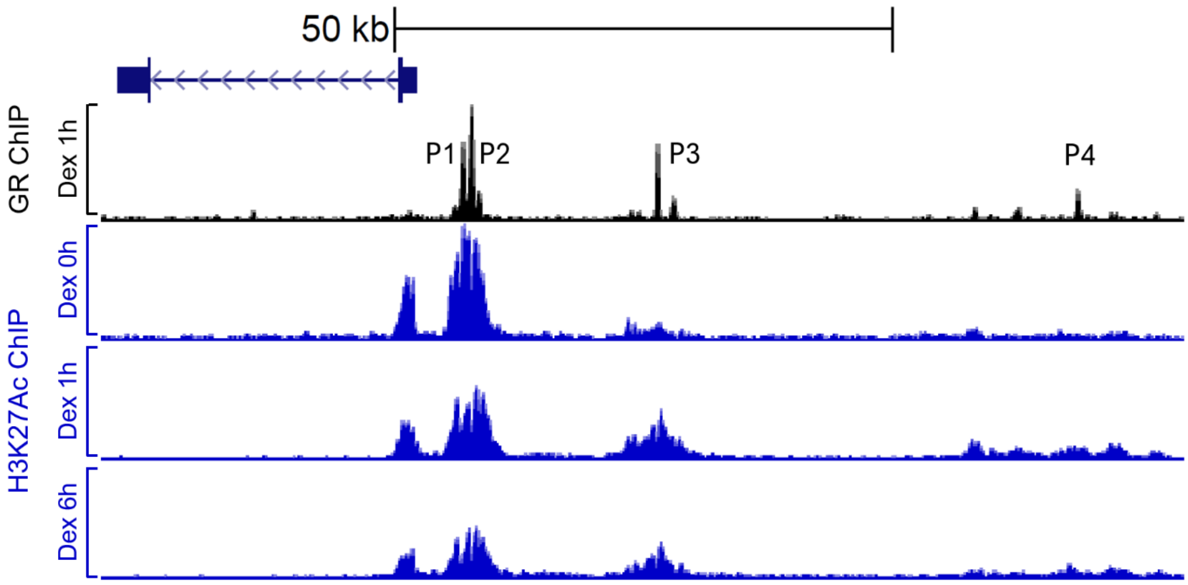

Figure S5. H3K27Ac marks near *KLF9* gene. Genome browser snapshot of *KLF9* gene along with ~70 kb of the 5′ upstream region showing ChIP-seq traces for GR (black) and H3K27Ac (blue) in A549 cells treated with 100 nM Dex for the indicated times are displayed (data from McDowell *et al.*, 2018 (24)). Positions of KLF9 GBSs P1-4 are indicated.

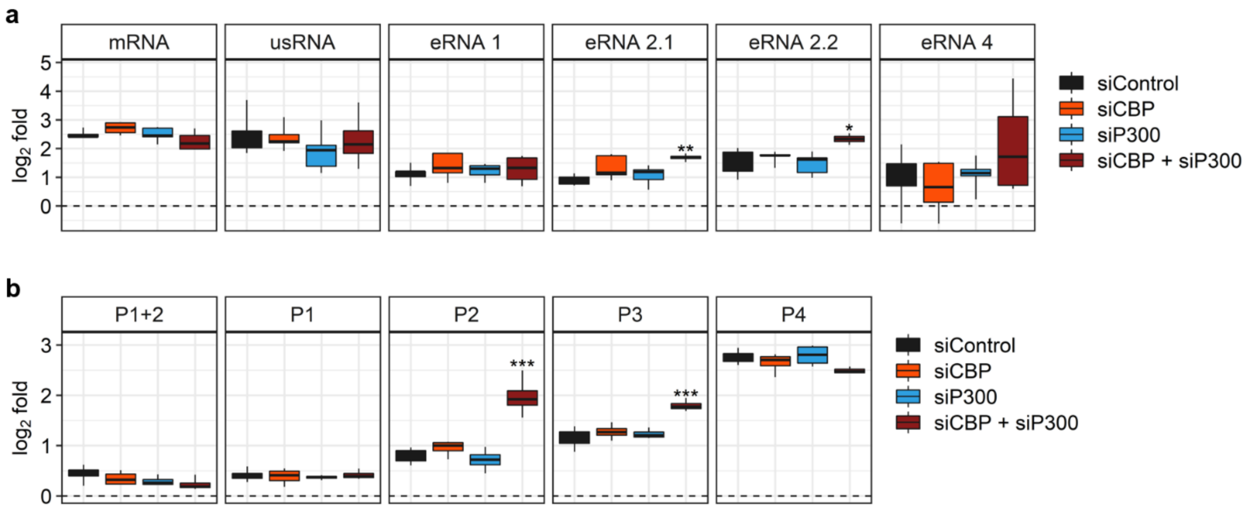

Figure S6. Effect of CBP and P300 knockdown on the glucocorticoid-mediated induction of KLF9 expression and enhancer activity from KLF9 GBSs. **a**, A549 cells were incubated with pools of 4 siRNAs, with overall concentration of 1 nM, for 48 h. The pools were either non-targeting siRNA (siControl; black), or siRNAs targeted to CBP (siCBP; orange), P300 (siP300; light blue), or combination of CBP and P300 siRNA (dark red). Cells were either not stimulated or treated with 300 nM budesonide for 6 h prior to qPCR analysis of: mature KLF9 mRNA, KLF9 usRNA, and the four eRNAs, 1, 2.1, 2.2, and 4. For normalization, GAPDH and U6 were also assayed. Normalized data (N = 4), expressed as mRNA/GAPDH, usRNA/U6, or eRNA/U6, were plotted as $\log_2$ fold change relative to no treatment control of each condition. **b**, A549 cells stably transfected with empty vector or reporter constructs for P1+P2(–) and P1 – P4(–) GBSs were treated with siRNAs and Bud as in (b) prior to luciferase assay. The RLU measurements were normalized to that of the empty vector for each condition (siControl, siCBP, siP300, or siCBP + siP300). Normalized data (N = 4), expressed as RLU/RLU_{empty}, were plotted as $\log_2$ fold change relative to no treatment control of each condition. Significance, using fold change relative to that of siControl group was tested using ANOVA with Tukey's post hoc test. * $P \leq 0.05$, ** $P \leq 0.01$, *** $P \leq 0.001$.

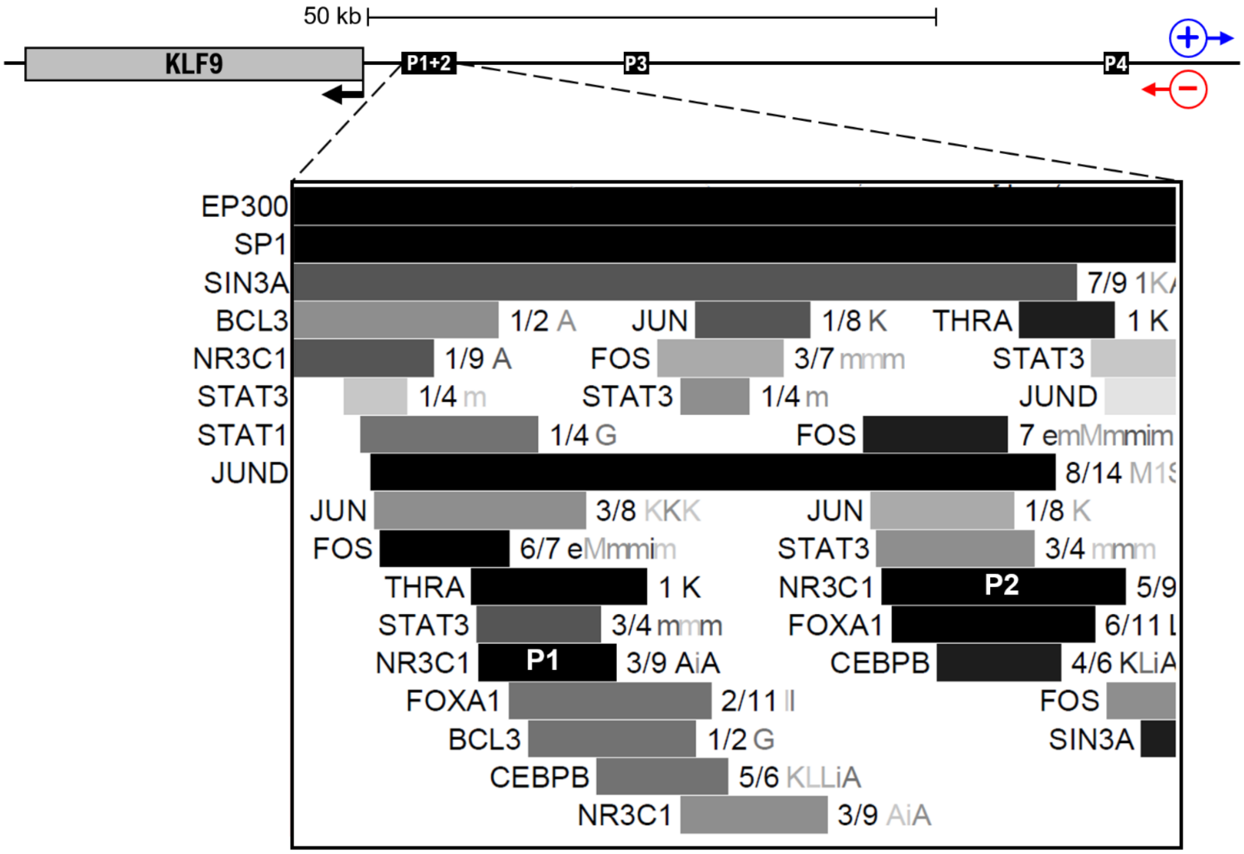

Figure S7. Transcription factor binding to the P1/P2 GBS. Schematic of the *KLF9* gene along with ~70 kb of the 5′ upstream region harboring the GBSs (black boxes) described in figure 3. Inset shows zoomed-in Genome Browser snapshots of ENCODE Transcription Factor ChIP-seq Clusters track for a select set of transcription factors binding traces at P1/P2 region (66). The intensity of the grey color is proportional to the maximum signal strength observed in any cell type contributing to each peak. The GR (NR3C1) ChIP peaks that are corresponding to P1 and P2 GBSs described in figure 3 are indicated.
